# P1245 Polymorphic Variants of HSD3B1 Gene Confer Different Outcome in Specific Subgroups of Patients Infected With SARS-CoV-2

**DOI:** 10.3389/fmed.2021.793728

**Published:** 2022-07-07

**Authors:** Samantha Epistolio, Giulia Ramelli, Margaret Ottaviano, Emanuele Crupi, Laura Marandino, Maira Biggiogero, Pier Andrea Maida, Lorenzo Ruinelli, Ursula Vogl, Dylan Mangan, Mariarosa Pascale, Marco Cantù, Alessandro Ceschi, Enos Bernasconi, Luca Mazzucchelli, Carlo Catapano, Andrea Alimonti, Christian Garzoni, Silke Gillessen Sommer, Federico Mattia Stefanini, Alessandra Franzetti-Pellanda, Milo Frattini, Ricardo Pereira Mestre

**Affiliations:** ^1^Laboratory of Molecular Pathology, Institute of Pathology, Ente Ospedaliero Cantonale, Locarno, Switzerland; ^2^Oncology Institute of Southern Switzerland, Ente Ospedaliero Cantonale, Bellinzona, Switzerland; ^3^Clinic Research Unit, Clinica Luganese Moncucco, Lugano, Switzerland; ^4^Clinic of Internal Medicine and Infectious Diseases, Clinica Luganese Moncucco, Lugano, Switzerland; ^5^Informatics and Communication Technology, Ente Ospedaliero Cantonale, Bellinzona, Switzerland; ^6^Clinical Trial Unit, Ente Ospedaliero Cantonale, Lugano, Switzerland; ^7^Institute of Laboratory Medicine, Ente Ospedaliero Cantonale, Lugano, Switzerland; ^8^Division of Clinical Pharmacology and Toxicology, Institute of Pharmacological Sciences of Southern Switzerland, Ente Ospedaliero Cantonale, Lugano, Switzerland; ^9^Faculty of Biomedical Sciences, Università della Svizzera italiana, Lugano, Switzerland; ^10^Department of Clinical Pharmacology and Toxicology, University Hospital Zürich, Zürich, Switzerland; ^11^Division of Infectious Diseases, Department of Medicine, Ente Ospedaliero Cantonale, Università della Svizzera italiana, Lugano, Switzerland; ^12^Faculty of Medicine, Division of Biomedical Sciences, University of Geneva, Geneva, Switzerland; ^13^Faculty of Experimental Therapeutics, Institute of Oncology Research, Università della Svizzera italiana, Bellinzona, Switzerland; ^14^Department of Molecular Oncology, Institute of Oncology Research, Università della Svizzera italiana, Bellinzona, Switzerland; ^15^Department of Enviromental Science and Policy, Faculty of Science and Technology-ESP, University of Milan, Milan, Italy

**Keywords:** SARS-CoV-2, *HSD3B1* gene polymorphism, androgen receptor, direct sequencing, Likelihood-ratio tests

## Abstract

**Introduction:** Severe respiratory syndrome coronavirus 2 (SARS-CoV-2) uses the androgen receptor (AR), through ACE2 receptor and TMPRSS2, to enter nasal and upper airways epithelial cells. Genetic analyses revealed that *HSD3B1* P1245C polymorphic variant increases dihydrotestosterone production and upregulation of TMPRSS2 with respect to P1245A variant, thus possibly influencing SARS-CoV-2 infection. Our aim was to characterize the *HSD3B1* polymorphism status and its potential association with clinical outcomes in hospitalized patients with COVID-19 in Southern Switzerland.

**Materials and Methods:** The cohort included 400 patients hospitalized for COVID-19 during the first wave between February and May 2020 in two different hospitals of Canton Ticino. Genomic DNA was extracted from formalin-fixed paraffin-embedded tissue blocks, and *HSD3B1* gene polymorphism was evaluated by Sanger sequencing. Statistical associations were verified using different test.

**Results:** *HSD3B1* polymorphic variants were not associated with a single classical factor related to worse clinical prognosis in hospitalized patients with SARS-CoV-2. However, in specific subgroups, *HSD3B1* variants played a clinical role: intensive care unit admission was more probable in patients with P1245C diabetes compared with P1245A individuals without this comorbidity and death was more associated with hypertensive P1245A>C cases than patients with P1245A diabetes without hypertension.

**Discussion:** This is the first study showing that *HSD3B1* gene status may influence the severity of SARS-CoV-2 infection. If confirmed, our results could lead to the introduction of *HSD3B1* gene status analysis in patients infected with SARS-CoV-2 to predict clinical outcome.

## Introduction

The spectrum of symptomatic infection caused by severe respiratory syndrome coronavirus 2 (SARS-CoV-2) ranges from mild or asymptomatic to severe illness with patients developing serious symptoms (dyspnea, hypoxia) requiring hospitalization (1). Unfortunately, after hospitalization about 5% of patients present with a critical illness (respiratory failure, shock, or multiorgan dysfunction) requiring admission to an intensive care unit (ICU) (1). Severe outcome after SARS-CoV-2 infection can arise in otherwise healthy individuals of any age, but predominantly occurs in adult men with advanced age and in patients with underlying medical comorbidities, such as hypertension, obesity, diabetes, cardiovascular disease, chronic respiratory disease, malignancies, and kidney disease (2–4).

To facilitate risk-stratification and management of coronavirus 2019 (COVID-19), a better understanding of the factors associated with severe disease is necessary. In particular, several studies have focused on the androgen pathway to explain differences between men and women (5, 6). Indeed, it has been recognized that SARS-CoV-2 binds to ACE2 to enter epithelial cells of the upper and lower respiratory tracts (7). Proteolytic cleavage of the viral SPIKE protein by TMPRSS2 allows the fusion of viral and cellular membranes. TMPRSS2 is a member of the family of type II transmembrane serine proteases that are implicated in several physiological and pathological processes, such as cancer and viral infections, including influenza A viruses, SARS-CoV, and MERS-CoV coronaviruses (7). TMPRSS2 has been widely studied in relation to prostate cancer and androgen receptor (AR) pathway because its expression is positively regulated by androgens through direct transcriptional regulation by AR (5, 8). Therefore, AR activity is considered a requirement for the transcription of TMPRSS2 gene as no other known TMPRSS2 gene promoter has been described in humans to date (9).

Many clinical parameters have been associated with SARS-CoV-2 infection severity, but nowadays there are no definitive genetic data supporting the role of androgen pathway in this viral infection (2–4). With regard to this point, the androgen dehydroepiandrosterone (DHEA) pathway may be relevant. This hormone and its sulfate (DHEA-S) are secreted by the adrenal gland, and peripherally, DHEA is metabolized by the enzyme 3β-hydroxysteroid dehydrogenase-1 (3β-HSD1; encoded by the *HSD3B1* gene) into downstream androgens (testosterone and dihydrotestosterone) (10, 11). There are two common germline missense encoding alleles of *HSD3B1* gene. The P1245A allele encodes for an adrenal restrictive enzyme that is rapidly degraded, thus limiting conversion from DHEA to downstream androgens leading to a downregulation of TMPRSS2 activity. In contrast, the P1245C allele encodes for an adrenal permissive enzyme that is resistant to degradation through ubiquitination mechanism, thus resulting in increased conversion of DHEA into more androgens and consequently to an upregulation of TMPRSS2 (12, 13). Among all the aforementioned clinical parameters associated with worse outcome in patients with SARS-CoV-2, the HSD3B1 polymorphism is associated only with hypertension and aldosterone level. Specifically, P1245A allele inheritance is associated with worsened outcomes in asthma as assessed by multiple clinical measures and absolute neutrophil count (10, 11).

Based on a possible influence of the AR pathway on SARS-CoV-2 infection severity, our aim was to explore the potential association between the *HSD3B1* gene polymorphism and clinical outcome in a series of patients who underwent hospitalization due to COVID-19 in Canton Ticino (Switzerland).

## Materials and Methods

### Study Population

This is an observational retrospective study conducted in two hospitals in Southern Switzerland that regularly shared clinical protocols during the pandemic phase (Ente Ospedaliero Cantonale, EOC; Clinica Luganese Moncucco). The study included patients hospitalized for SARS-CoV-2 infection during the first wave from February 29, 2020 to May 22, 2020, with the last discharge on June 12, 2020. Inclusion criteria were as follows: age greater than or equal to 18 years, the presence of positive SARS-CoV-2 PCR, clinical data and also age and sex details recorded, and availability of archival tissue samples (any type) collected at any time before SARS-CoV-2 infection for any clinical reason. Clinico-pathological characteristics of the SARS-CoV-2 infection and the clinical outcomes of interest were extracted from the EOC and Clinica Luganese Moncucco databases. For each case, informative data (i.e., sex, age, body mass index (BMI), and comorbidities) and clinical information [symptoms at presentation, days of hospitalization, ICU admission, death, and WHO (World Health Organization) scale of COVID-19 clinical improvement] were collected. In particular, comorbidities analyzed in our study were as follows: diabetes, cardiovascular diseases, hypertension, chronic respiratory disease, and tumor.

This study was approved by the regional ethics committee (reference number: TI3710, BASEC 2020-01801). For patients who were alive at study entry, an informed consent was required to be included in the project. The study was conducted in accordance with the Declaration of Helsinki.

### Characterization of P1245 Polymorphism in *HSD3B1* Gene

The analysis of *HSD3B1* gene was performed at the Institute of Pathology in Locarno (Switzerland). Genomic DNA was extracted from six 4-μm-thick serial sections of formalin-fixed paraffin-embedded tissue block, following the QIAGEN protocol (QIAamp DNA FFPE Tissue Kit 50, QIAGEN, Chatsworth, CA, USA).

The polymorphism of *HSD3B1* gene (codon 1245) was evaluated by direct sequencing. In total, 50 ng of genomic DNA was amplified using H_2_O, 1X PCR buffer (Applied biosystems, Foster City, CA, USA), 3.0 mM MgCl2 (Applied biosystems), 0.2 mM deoxyribonucleotides triphosphate (dNTPs) (GE Healthcare, Chicago, IL, USA), 2 U/s Taq Gold (Applied Biosystems), and 0.5μM of the respective HSD3B1 forward and reverse primers (forward: 5′-GTCAAATAGCGTATTCACCTTCTCTTAT-3′; reverse: 5′-GAGGGTGGAGCTTGATGACATCT-3′) (12), in a 23 μl PCR. PCR was performed applying the following thermal profile: 1 cycle for 2′ at 50°C; 1 cycle for 10′at 95°C; 40 cycles constituted of 15′′ at 95°C (denaturation), 30′′ at 67°C (annealing), 30′′ at 72°C (extension); 1 cycle for 3′ at 72°C; 10°C hold. Samples were subjected to automated sequencing on SeqStudio Genetic Analyzer (Applied Biosystems), and the sequences were evaluated by Sequencing Analysis Software 7 (Applied Biosystems). After direct sequencing, the cases were distinguished, based on *HSD3B1* polymorphism, in three groups: P1245A (homozygous A), P1245C (homozygous C), and P1245A>C (heterozygous).

### Statistical Analyses

Summary statistics for clinical data, comorbidities, age, sex, BMI, and *HSD3B1* polymorphism were calculated on each variable and for pairs of variables, such as the relative chi-squared test and the Pearson's correlation between two variables. Typical summaries such as mean, standard deviation, and relative frequencies were also calculated on conditional distributions, for example, death given sex. Several statistical tests were applied to find hypotheses deserving further medical investigation. Statistical hypotheses were tested by Kruskal–Wallis rank sum test, asymptotic Wilcoxon–Mann–Whitney test, Mood two-sample test of scale, and asymptotic two-sample Fisher-Pitman permutation test after obtaining marginal and bivariate summary statistics for clinical data, comorbidities, age, sex, BMI, and *HSD3B1* polymorphism (14, 15). Generalized linear models were also fitted to characterize the dependence of a variable of interest on several other explanatory variables. Likelihood-ratio tests were performed to assess the contribution of a model term, as the interaction among two explanatory variables X by W, given all other terms already included into the model, say X and W; Wald statistical tests were performed on the null hypothesis that a parameter associated with a variable is equal to zero (16). All computations were performed using the R software (17).

## Results

### Patients' Characteristics

The study cohort included 400 patients; 234 men (58.50%) and 166 women (41.50%) hospitalized for COVID-19 during the first wave. The mean age at hospitalization was 70.95 years (range: 29–98 years), and the mean hospitalization duration was 14 days (Supplementary Table S1).

Considering the whole cohort, 18.04% (72/399) of patients accessed ICU and 19.75% (79/400) died (Supplementary Table S1). The mean stay in ICU was 2.68 days, with a minimum of 1 day and a maximum of 67 days; in particular, 32 cases (32/397, 8.06%) stayed in ICU for at least 10 days (Supplementary Table S1).

The most common symptom associated with SARS-CoV-2 infection was fever (294/398, 73.87%) followed by respiratory (286/398, 71.86%) and gastrointestinal problems (69/398, 17.34%). Concerning respiratory complications, pneumonia was diagnosed in 70.93% of patients (283/399).

Comorbidities were reported in 74% of the study population (296/400) with 45.50% of the cohort presenting more than one comorbidity (182/400) (Supplementary Table S1). In particular, the individuals with diabetes were 102 (25.50%), with cardiovascular diseases 145 (36.25%), hypertension 208 (52%), chronic respiratory disease 80 (21.56%), and malignancies 61 (15.25%). The BMI value was available for 341 patients and, out of them, 94 cases (27.57%) presented a value greater than or equal to 30, corresponding to obesity (Supplementary Table S1). Evaluation of the WHO scale of clinical improvement was available for 323 cases and 49 (15.17%) out of these patients presented high values (from 5 to 8) at presentation (Supplementary Table S1).

### Evaluation of *HSD3B1* Polymorphism

Molecular characterization of the *HSD3B1* gene polymorphism was evaluable in all the 400 cases included in the study. In particular, the P1245A polymorphic variant was described in 158 cases (158/400, 39.50%), P1245C in 55 individuals (55/400, 13.75%), and P1245A>C in 187 samples (187/400, 46.75%) (Supplementary Figure S1 and Supplementary Table S1). Overall, 345 out of 400 patients had at least an A allele (86.25%), whereas cases with at least a C allele were 242 out of 400, corresponding to 60.50% (Supplementary Table S1).

### Bivariate Summaries of Marginal Distributions

All the variables were matched against another one with bivariate associations applying the chi-squared test (Supplementary Table S2): here, we report only those that showed statistical significance or those that are the focus of our study. Among the 400 patients admitted in hospital for SARS-CoV-2 infection, men were more likely to have ICU admission (*p* = 0.00038), fever (*p* = 0.0348), cardiovascular disease as comorbidity (*p* = 0.0242), and to be classified on a high WHO scale grade, ranging from 5 to 8 (*p* = 0.0349).

In addition, regardless of gender, death was associated with admission to ICU (*p* < 1*10^−4^) and with the presence of comorbidities (specifically diabetes, *p* = 0.03; cardiovascular disease, *p* < 1*10^−4^; hypertension, *p* = 0.0179; and malignancy, *p* = 0.00315). The occurrence of respiratory symptoms and pneumonia in patients infected with SARS-CoV-2 were associated with ICU admission (*p* = 0.0339 and *p* = 0.015, respectively) but these factors, in addition to chronic respiratory diseases, were not correlated with death. ICU admission was also linked to diabetes and to high WHO scale grade (with *p* = 0.0343 and *p* < 1*10^−4^, respectively). The majority of hospitalized patients with diabetes also suffered from hypertension and had a BMI ≥ 30 (*p* < 1*10^−4^ and *p* = 0.00456, respectively). In addition, cardiovascular diseases were associated with the presence of hypertension (*p* < 1*10^−4^) and hypertension with BMI ≥ 30 (*p* = 0.043). Finally, BMI ≥ 30 was a feature of cases with higher WHO scale grade (*p* = 0.00218), and high BMI was associated with patients presenting more than one comorbidity (*p* = 0.0443).

*HSD3B1* gene polymorphic status was not associated with any clinical parameter in bivariate associations in the overall population (Supplementary Table S3).

### Correlations Inside Subgroups of Clinical Data

The Kruskal–Wallis rank sum test, the asymptotic Wilcoxon–Mann–Whitney test, the Mood two-sample test of scale, and the asymptotic two-sample Fisher-Pitman permutation test, applied after obtaining marginal and bivariate summary statistics, did not demonstrate any statistically significant correlation for clinical data, comorbidities, age, sex, BMI, and *HSD3B1* polymorphism correlations inside subgroups.

### Correlations Between Variables With Respect to ICU Admission

**Figure 1:**
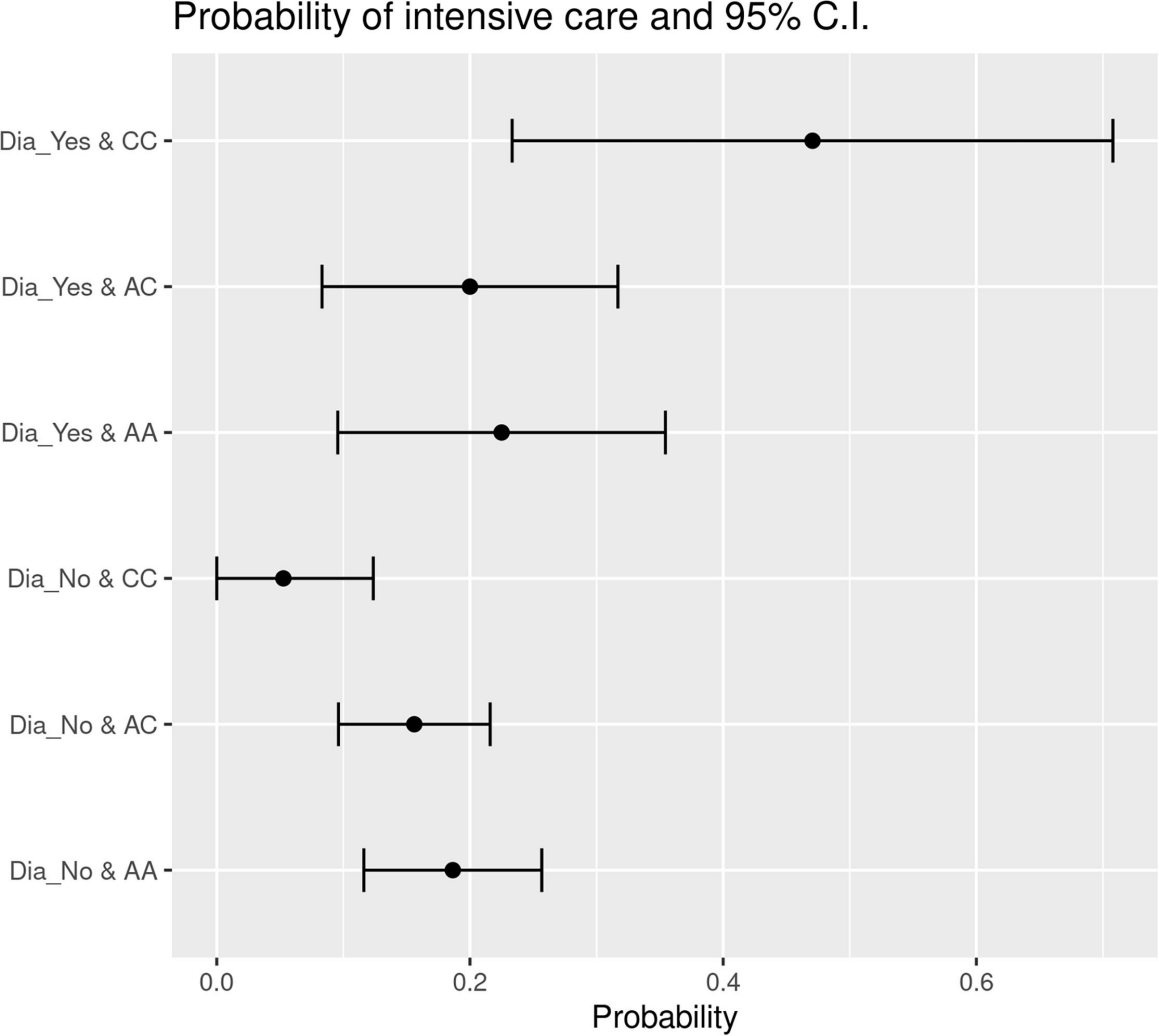
Probability of ICU entrance subdivided based on the presence of diabetes in the different *HSD3B1* polymorphism variants. In each specific polymorphic variant subgroup, the presence of diabetes increases the probability of ICU entrance. P1245C is the one with higher increment, showing an increase of 8.9 times in the presence of diabetes. AA, P1245A polymorphic variant; AC, P1245A>C polymorphic variant; CC, P1245C polymorphic variant; CI, confidence interval; Dia_Yes, the presence of diabetes; Dia_No, the absence of diabetes.

The application of Likelihood-ratio tests and Wald statistical tests permitted to define that *HSD3B1* polymorphism subgroups status was significantly associated with diabetes concerning ICU admission. The probability of ICU entrance for P1245C diabetic individuals was 47.10% whereas for the ones without this comorbidity was only 5.26%. On the contrary, within the individuals characterized by homozygous P1245A status, the probability of ICU admission was 22.50% for patients with diabetes and 18.63% for cases without this comorbidity (Figure 1). In addition, in the group of patients with a *HSD3B1* heterozygous profile (P1245A>C), the probability corresponded to 20% for patients with diabetes and 15.60% for patients without diabetes (Figure 1). Overall, patients with P1245A and P1245A>C had a similar probability of ICU admission regardless of the presence of diabetes as comorbidity (1.21- and 1.28-fold increase, respectively), whereas in the P1245C group, this probability increased 8.95 times in the presence of diabetes. Finally, by comparing the different subgroups, the only statistically significant association was reported between P1245C in patients with diabetes with respect to patients with P1245A nondiabetes, which were more prone to have ICU admission compared with P1245A without this comorbidity (*p* = 0.00976).

The same result was obtained comparing the patients without diabetes and at least one A allele (i.e., P1245A or P1245A>C) and the patients with P1245C and diabetes (*p* = 0.00707). In these subgroups, the probability of ICU admission was more than 10 times higher for patients with P1245C diabetes if compared to patients P1245A nondiabetes (27.53 vs. 2%).

Furthermore, ICU admission was found to be influenced by sex and polymorphism. More in details, P1245C men were more prone to have ICU admission compared with P1245A+P1245A>C women (*p* = 0.00635).

### Correlations Between Variables With Respect to Death

**Figure 2:**
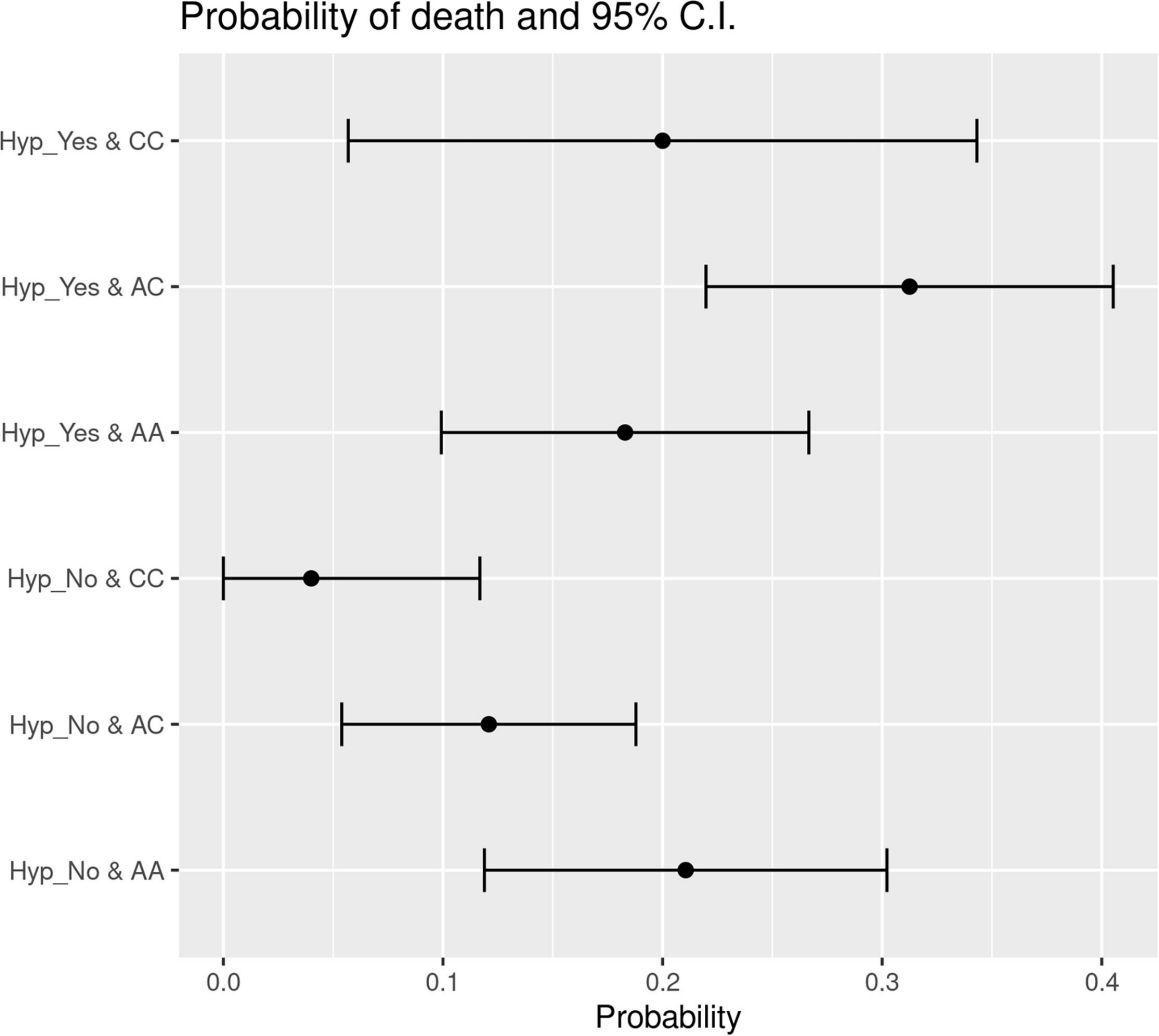
Probability of death subdivided based on the presence of hypertension in the different *HSD3B1* polymorphism variants. In both P1245A>C and P1245C variants, the presence of hypertension increases the probability of decease; instead, P1245A cases present the opposite trend. P1245C is the one with higher increment, showing an increase of 5 times in the presence of hypertension. AA, P1245A polymorphic variant; AC, P1245A>C polymorphic variant; CC, P1245C polymorphic variant; CI, confidence interval; Hyp_Yes, the presence of hypertension; Hyp_No, the absence of hypertension.

The data obtained applying Likelihood-ratio tests and Wald statistical tests indicate that *HSD3B1* polymorphism subgroups' status was significantly associated with hypertension in patients who died. The probability of decease was 31.25% for hypertensive P1245A>C cases and only 12.09% for P1245A>C patients without hypertension. With regard to the P1245A variant, the probability of death was 18.29% for cases with hypertension and 21.05% for nonhypertensive cases. In P1245C subgroup, the probability of death was 20% in hypertensive patients and only 4% in individuals without hypertension as comorbidity (Figure 2). Overall, in the group of P1245A patients, there was no difference in the probability of death concerning the presence or absence of hypertension. On the contrary, hypertension (if compared to absence of this comorbidity) conferred a higher death probability of 2.58 and five times in the group of P1245A>C and P1245C, respectively (Figure 2). Between these subgroups, hypertensive P1245A>C patients were significantly more prone to die than patients characterized by P1245A and without this comorbidity (*p* = 0.0142). A similar trend was observed when hypertensive P1245C patients were compared with P1245A patients without hypertension (*p* = 0.0977).

In addition, with respect to the risk of death, there was a significant association between *HSD3B1* polymorphism subtype and patients' age. Indeed, we observed a direct correlation between the linear increase of age and the increased possibility of death in the group of patients characterized by P1245C genotype, if compared to patients with other *HSD3B1* gene status (P1245A and P1245A>C) (*p* = 0.03467).

## Discussion

There are well-known parameters leading to worse outcome in patients infected with SARS-CoV-2, such as older age, male gender, high BMI, or the presence of at least one comorbidity among diabetes, cardiovascular diseases, hypertension, chronic respiratory diseases, and malignancy. However, we must keep in mind that the disease course is different among patients and its severity ranges dramatically from asymptomatic to multiorgan failure, based on individual features (18, 19). This different trend of COVID-19 outcome led to hypothesize a putative clinical relevance of patients' genetic status. Indeed, there is a growing body of evidence that the individual course of SARS-CoV-2 infection, in terms of susceptibility, severity, and overall clinical outcomes, may be influenced by genetic factors (20–22). Starting from this concept, we decided to investigate the role of the *HSD3B1* gene as a consequence of its involvement in the AR pathway, the preferred way of SARS-CoV-2 infection in humans. In addition, this might explain the well-documented sex differences in the course of the disease (6, 10, 11, 23). In our cohort, that included hospitalized patients living in Southern Switzerland, the frequencies of *HSD3B1* P1245A, P1245A>C, and P1245C were similar to the data already published in the literature regarding the Caucasian population (24, 25). The representativeness of our study was also substantiated by the confirmation of the aforementioned parameters linked to worse outcome after SARS-CoV-2 infection (2–4, 26–28). When we investigated the role of *HSD3B1* polymorphic variants, we did not observe any correlation in bivariate analysis in the whole population leading to a lack of association between P1245 and hospitalization. However, focusing on statistical correlations in groups of patients with specific clinical characteristics, we obtained interesting results concerning the influence of *HSD3B1* in patients infected with SARS-CoV-2. In particular, we focused on ICU admission and possibility of death, founding that HSD3B1 synergizes with specific clinical parameters.

With regard to ICU admission, we observed that patients characterized by P1245A or P1245A>C were more or less likely to enter ICU, with a small increase for patients with diabetes. For P1245C, the difference was very marked, with an increase from 5.26 to 47.1% when patients with diabetes were compared with those who were not characterized by this comorbidity. Furthermore, a significant association was reported when patients with P1245C diabetes were compared to patients with P1245A nondiabetes. Therefore, our data confirm that diabetes is a general factor of worse course of SARS-CoV-2 infection, but it seems that *HSD3B1* gene status may contribute: indeed, the increase of probability of ICU admission increases 9-fold in P1245C patients, showing a synergistic effect of diabetes and *HSD3B1* gene status.

In addition, P1245C men were more predisposed to ICU entrance compared with P1245A+P1245A>C women. This notion can further support the explanation of the worst COVID-19 prognosis demonstrated in men, as described before.

When we looked at probability of death, we observed a similar association between the C allele of *HSD3B1* and hypertension. Indeed, the probability of death was similar in P1245A, regardless of the presence of hypertension. However, when P1245A>C or P1245C were taken into account, this value showed a 2.5- and 5-fold increase, respectively. Moreover, if compared to patients with nonhypertensive P1245A, P1245A>C and, borderline, P1245C (the last finding probably due to size cohort, being the P1245C less represented) had a significantly higher probability of death. In addition, also in this case, we can see that the higher risk of death, that hypertension confers to patients infected with SARS-CoV-2, seems to be driven by *HSD3B1* genetic status.

The last significance defined in the statistical models of our study is the direct correlation between the linear increase of age and the increased possibility of death in the group of patients characterized by P1245C genotype, with respect to P1245A and P1245A>C. This evidence suggests that P1245C cases have a higher probability of death as age increases in comparison to the other subgroups of patients. As a consequence, in the daily clinical practice, the treatment of older P1245C individuals may indicate the need of more aggressive treatments against infection.

The aforementioned findings reinforce the role played by the AR pathway in SARS-CoV-2 infection, suggesting that a more active pathway (observed when at least a C allele is present) directly influences the severity of the course of such infection. The relationship between AR pathway and diabetes or hypertension deserves further investigation because, to the best of our knowledge, the role played by *HSD3B1* with these comorbidities has never been investigated. More importantly, our data may permit to distinguish, in the group of patients characterized by a specific comorbidity, a particular subclass with higher risk of ICU entrance or death through the evaluation of only a gene polymorphism using a relatively fast and cost-effective methodology (i.e., direct sequencing).

The main limitation of this study is the size cohort. The investigation of the correlation between *HSD3B1* polymorphic variants and all the parameters known to be clinically relevant in SARS-CoV-2 infection would require a significant greater number of patients, which unfortunately are not available in our region (Canton Ticino). A possible solution to overcome this problem could be the enlargement of the study population. However, this extension could lead to the introduction of new variables that should be taken in account (e.g., the different therapies that were not available in the first months of 2020 for the care of SARS-CoV-2 infection) and that may have an additional influence on the course of disease, masking the strengthening effect played by genetic variants.

In conclusion, our study suggests that genetic status of patients may play a role in SARS-CoV-2 infection, indicating the *HSD3B1* P1245 polymorphism as a potential new marker in case of specific comorbidities, to better predict the course of disease and, as a consequence, to intensify the therapies based on the patients' genetic status of the AR pathway. Further validation of these results in a larger population of patients is needed.

## Data Availability Statement

The datasets presented in this study can be found in the online repository open science framework (OSF) repository following the link: https://osf.io/g7mzr/?view_only=1c120cab63d3492a98a6418d4612c6e7 or in the Supplementary Material.

## Ethics Statement

The studies involving human participants were reviewed and approved by Regional Ethics Committee (Ref. number: TI3710, BASEC 2020-01801). The patients/participants provided their written informed consent to participate in this study.

## Author Contributions

SE, GR, MO, LMar, MF, and RP wrote the first draft of the manuscript. RP, MO, LMar, EC, MB, PM, AF-P, LR, UV, MP, MC, AC, EB, CC, AA, and CG selected the cohort for the analyses. SE and GR performed all the molecular characterization. SE, GR, and MF evaluated direct sequencing results. DM and FS performed statistical analyses. SE, GR, MF, and RP prepared the final version of the manuscript. SG, LMaz, MF, and RP supervised the whole project. All authors contributed to the article and approved the submitted version.

## Funding

This study was completely supported by the scientific funds from Ente Ospedaliero Cantonale (EOC) to Dr. Med. Pereira Mestre Ricardo.

## Conflict of Interest

The authors declare that the research was conducted in the absence of any commercial or financial relationships that could be construed as a potential conflict of interest.

## Publisher's Note

All claims expressed in this article are solely those of the authors and do not necessarily represent those of their affiliated organizations, or those of the publisher, the editors and the reviewers. Any product that may be evaluated in this article, or claim that may be made by its manufacturer, is not guaranteed or endorsed by the publisher.

## Abbreviations

AR: androgen receptor
BMI: body mass index
COVID-19: coronavirus 2019
DHEA: androgen dehydroepiandrosterone
EOC: Ente Ospedaliero Cantonale
ICU: intensive care unit
P1245A: *HSD3B1* homozygous A (codon 1245)
P1245C: *HSD3B1* homozygous C (codon 1245)
P1245A>C: *HSD3B1* heterozygous A>C (codon 1245)
SARS-CoV-2: severe respiratory syndrome coronavirus 2
WHO: World Health Organization
3β-HSD1: 3β-hydroxysteroid dehydrogenase-1.

## References

[1] Wu Z McGoogan JM. Characteristics of and important lessons from the coronavirus disease 2019 (COVID-19) outbreak in China: summary of a report of 72 314 cases from the Chinese center for disease control and prevention. JAMA. (2020) 323:1239–42. 10.1001/jama.2020.2648 32091533

[2] Grasselli G Zangrillo A Zanella A Antonelli M Cabrini L Castelli A. Baseline characteristics and outcomes of 1591 patients infected with SARS-CoV-2 admitted to ICUs of the Lombardy Region. Italy. (2020) 323:1574–81. 10.1001/jama.2020.5394 32250385 PMC7136855

[3] Richardson S Hirsch JS Narasimhan M Crawford JM McGinn T Davidson KW. Presenting characteristics, comorbidities, and outcomes among 5700 patients hospitalized with COVID-19 in the New York City area. JAMA. (2020) 323:2052–9. 10.1001/jama.2020.6775 32320003 PMC7177629

[4] Zhou F Yu T Du R Fan G Liu Y Liu Z. Clinical course and risk factors for mortality of adult inpatients with COVID-19 in Wuhan, China: a retrospective cohort study. Lancet. (2020) 395:1054–62. 10.1016/S0140-6736(20)30566-3 32171076 PMC7270627

[5] Cheng J Zhou J Fu S Fu J Zhou B Chen H. Prostate adenocarcinoma and COVID-19: the possible impacts of TMPRSS2 expressions in susceptibility to SARS-CoV-2. J Cell Mol Med. (2021) 25:4157–65. 10.1111/jcmm.16385 33609069 PMC8013364

[6] Rehman S Ravinayagam V Nahvi I Aldossary H Al-Shammari M Amiri MSA. Immunity, sex hormones, and environmental factors as determinants of COVID-19 disparity in women. Front Immunol. (2021) 12:680845. 10.3389/fimmu.2021.680845 34484179 PMC8416472

[7] Hoffmann M Kleine-Weber H Schroeder S Krüger N Herrler T Erichsen S. SARS-CoV-2 cell entry depends on ACE2 and TMPRSS2 and is blocked by a clinically proven protease inhibitor. Cell. (2020) 181:271–80. 10.1016/j.cell.2020.02.052 32142651 PMC7102627

[8] Semaan L Mander N Cher ML Chinni SR. TMPRSS2-ERG fusions confer efficacy of enzalutamide in an in vivo bone tumor growth model. BMC Cancer. (2019) 19:972. 10.1186/s12885-019-6185-0 31638934 PMC6802314

[9] National Institutes of Health. TMPRSS2 transmembrane serine protease 2 [Homo sapiens (human)] (2020). Gene ID: 7113. Available online at : https://www.ncbi.nlm.nih.gov/gene/7113 (accessed September 06, 2021).

[10] Shimodaira M Nakayama T Sato N Aoi N Sato M Izumi Y. Association of HSD3B1 and HSD3B2 gene polymorphisms with essential hypertension, aldosterone level, and left ventricular structure. Eur J Endocrinol. (2010) 163:671–80. 10.1530/EJE-10-0428 20660004

[11] Zein J Gaston B Bazeley P DeBoer MD Igo RPJr Bleecker ER. HSD3B1 genotype identifies glucocorticoid responsiveness in severe asthma. Proc Natl Acad Sci USA. (2020) 117:2187–93. 10.1073/pnas.1918819117 31932420 PMC6995013

[12] Hearn JWD AbuAli G Reichard CA Reddy CA Magi-Galluzzi C Chang KH. HSD3B1 and resistance to androgen-deprivation therapy in prostate cancer: a retrospective, multicohort study. Lancet Oncol. (2016) 17:1435–44. 10.1016/S1470-2045(16)30227-3 27575027 PMC5135009

[13] Hettel D Sharifi N. HSD3B1 status as a biomarker of androgen deprivation resistance and implications for prostate cancer. Nat Rev Urol. (2018) 15:191–6. 10.1038/nrurol.2017.201 29231195

[14] Hollander M Wolfe DA. Nonparametric Statistical Methods. New York: John Wiley and Sons (1973).

[15] Conover WJ. Practical Nonparametric Statistics. New York: John Wiley and Sons (1971).

[16] Dobson AJ. An Introduction to Generalized Linear Models. London: Champan and Hall (1990).

[17] R Core Team. A Language and Environment For Statistical Computing. R Foundation for Statistical Computing. (2020). Available online at: https://www.R-project.org/. (accessed September 20, 2021).

[18] Guan WJ Ni ZY Hu Y Liang WH Ou CQ He JX. Clinical Characteristics of Coronavirus Disease 2019 in China. N Engl J Med. (2020) 382:1708–20. 10.1056/NEJMoa2002032 32109013 PMC7092819

[19] Berlin DA Gulick RM Martinez FJ. Severe Covid-19. N Engl J Med. (2020) 383:2451–60. 10.1056/NEJMcp2009575 32412710

[20] Ovsyannikova IG Haralambieva IH Crooke SN Poland GA Kennedy RB. The role of host genetics in the immune response to SARS-CoV-2 and COVID-19 susceptibility and severity. Immunol Rev. (2020) 296:205–19. 10.1111/imr.12897 32658335 PMC7404857

[21] Severe Covid-19 GWAS Group: Ellinghaus D Degenhardt F Bujanda L Buti M Albillos A. Genomewide association study of severe covid-19 with respiratory failure. N Engl J Med. (2020) 383:1522–34. 10.1056/NEJMoa2020283 32558485 PMC7315890

[22] COVID-19 Host Genetics Initiative. The COVID-19 host genetics initiative, a global initiative to elucidate the role of host genetic factors in susceptibility and severity of the SARS-CoV-2 virus pandemic. Eur J Hum Genet. (2020) 28:715–8. 10.1038/s41431-020-0636-6 32404885 PMC7220587

[23] Jin JM Bai P He W Wu F Liu XF Han DM. gender differences in patients with COVID-19: focus on severity and mortality. Front Public Health. (2020) 8:152. 10.3389/fpubh.2020.00152 32411652 PMC7201103

[24] Ntostis P Peraki O Boulgari A Agiannitopoulos K Pantos K Lamnissou K. Genetic variation in the HSD3B1 gene and recurrent spontaneous abortions. J Matern Fetal Neonatal Med. (2012) 25:408–10. 10.3109/14767058.2011.582199 21631238

[25] Khalaf DJ Aragón IM Annala M Lozano R Taavitsainen S Lorente D. HSD3B1 (1245A>C) germline variant and clinical outcomes in metastatic castration-resistant prostate cancer patients treated with abiraterone and enzalutamide: results from two prospective studies. Ann Oncol. (2020) 31:1186–97. 10.1016/j.annonc.2020.06.006 32574722

[26] Wang B Li R Lu Z Huang Y. Does comorbidity increase the risk of patients with COVID-19: evidence from meta-analysis. Aging. (2020) 12:6049–57. 10.18632/aging.103000 32267833 PMC7185114

[27] Brar G Pinheiro LC Shusterman M Swed B Reshetnyak E Soroka O. COVID-19 severity and outcomes in patients with cancer: a matched cohort study. J Clin Oncol. (2020) 38:3914–24. 10.1200/JCO.20.01580 32986528 PMC7676890

[28] Gao M Piernas C Astbury NM Hippisley-Cox J O'Rahilly S Aveyard P. Associations between body-mass index and COVID-19 severity in 6·9 million people in England: a prospective, community-based, cohort study. Lancet Diabetes Endocrinol. (2021) 9:350–9. 10.1016/S2213-8587(21)00089-9 33932335 PMC8081400

