# Supplementary material for: P1245 Polymorphic Variants of HSD3B1 Gene Confer Different Outcome in Specific Subgroups of Patients Infected With SARS-CoV-2
Source: Front Med (Lausanne). 2022 Jul 7;8:793728. doi: 10.3389/fmed.2021.793728 (PMC9302441; doi:10.3389/fmed.2021.793728)
Supplement: Supplementary file 1 [file Table_1.DOCX]

| **N** | **Sex** | **Age** | **BMI** | **WHO scale** | **Hospitalization**  **date** | **Discharge**  **date** | **Death** | **ICU** | **ICU**  **(days)** | **COVID-19 symptoms** | | | **Confirmed pneumonia** | **Comorbidities** | | | | | ***HSD3B1***  **pol** |
| --- | --- | --- | --- | --- | --- | --- | --- | --- | --- | --- | --- | --- | --- | --- | --- | --- | --- | --- | --- |
|  |  |  |  |  |  |  |  |  |  | **Respiratory**  **symptoms** | **Fever** | **GI**  **symptoms** |  | **Diabetes** | **Cardiovascular Diseases** | **Hypertension** | **CRD** | **Tumor** |  |
| **1** | F | 91 | 27.97 | 8 | 29.02.2020 | 14.03.2020 | 1 | 0 | 0 | 1 | 1 | 0 | 1 | 0 | 1 | 1 | 0 | 0 | P1245A |
| **2** | M | 55 | 40.96 | 6 | 01.03.2020 | 27.03.2020 | 0 | 1 | 11 | 1 | 1 | 0 | 0 | 1 | 0 | 1 | 1 | 0 | P1245C |
| **3** | M | 62 | 28.66 | 5 | 04.03.2020 | 31.03.2020 | 0 | 0 | 0 | 1 | 1 | 0 | 0 | 1 | 0 | 1 | 1 | 0 | P1245A>C |
| **4** | M | 78 | 29.60 | 8 | 05.03.2020 | 17.03.2020 | 1 | 1 | 4 | 0 | 1 | 0 | 1 | 1 | 1 | 0 | 0 | 0 | P1245A>C |
| **5** | F | 85 | 26.68 | 8 | 05.03.2020 | 07.04.2020 | 1 | 1 | 67 | 1 | 1 | 0 | 1 | 1 | 1 | 1 | 0 | 0 | P1245A |
| **6** | M | 46 | 28.88 | 7 | 05.03.2020 | 20.04.2020 | 0 | 1 | 39 | 1 | 1 | 0 | 0 | 0 | 0 | 0 | 0 | 0 | P1245A |
| **7** | M | 44 | 20.57 | 3 | 06.03.2020 | 07.05.2020 | 0 | 0 | 0 | 1 | 1 | 0 | 1 | 0 | 0 | 0 | 0 | 0 | P1245A>C |
| **8** | M | 76 | 26.40 | 3 | 07.03.2020 | 24.03.2020 | 0 | 1 | 2 | 1 | 1 | 0 | 0 | 0 | 0 | 1 | 0 | 0 | P1245A |
| **9** | F | 81 | 30.57 | 8 | 07.03.2020 | 17.03.2020 | 1 | 0 | 0 | 1 | 0 | 0 | 0 | 0 | 0 | 0 | 1 | 0 | P1245A |
| **10** | M | 67 | NA | 8 | 08.03.2020 | 14.03.2020 | 1 | 1 | 7 | 1 | 0 | 0 | 1 | 0 | 0 | 1 | 0 | 0 | P1245A |
| **11** | M | 78 | 23.55 | 3 | 08.03.2020 | 16.03.2020 | 0 | 0 | 0 | 1 | 1 | 0 | 0 | 0 | 0 | 1 | 0 | 0 | P1245A>C |
| **12** | M | 76 | 28.72 | 4 | 09.03.2020 | 24.03.2020 | 0 | 0 | 0 | 1 | 0 | 0 | 1 | 1 | 1 | 1 | 1 | 0 | P1245A |
| **13** | M | 46 | 27.00 | 3 | 09.03.2020 | 17.03.2020 | 0 | 0 | 0 | 1 | 1 | 1 | 1 | 0 | 0 | 0 | 0 | 0 | P1245A>C |
| **14** | M | 82 | 24.13 | 4 | 09.03.2020 | 20.03.2020 | 0 | 0 | 0 | 1 | 1 | 0 | 0 | 1 | 0 | 1 | 0 | 0 | P1245A>C |
| **15** | M | 78 | 25.70 | 8 | 09.03.2020 | 13.03.2020 | 1 | 0 | 0 | 1 | 1 | 0 | 1 | 0 | 1 | 1 | 0 | 1 | P1245A>C |
| **16** | F | 74 | 24.23 | 8 | 08.03.2020 | 20.03.2020 | 1 | 1 | 9 | 1 | 1 | 0 | 1 | 0 | 1 | 0 | 0 | 1 | P1245A |
| **17** | M | 37 | 39.19 | 4 | 09.03.2020 | 16.03.2020 | 0 | 0 | 0 | 1 | 1 | 0 | 0 | 0 | 0 | 0 | 0 | 0 | P1245A |
| **18** | M | 86 | 28.41 | 4 | 09.03.2020 | 02.04.2020 | 0 | 0 | 0 | 1 | 1 | 0 | 1 | 0 | 1 | 1 | 1 | 0 | P1245A>C |
| **19** | M | 74 | 22.14 | 4 | 10.03.2020 | 02.05.2020 | 0 | 0 | 0 | 1 | 0 | 0 | 0 | 0 | 1 | 0 | 1 | 0 | P1245A |
| **20** | M | 70 | 30.10 | 3 | 10.03.2020 | 20.03.2020 | 0 | 0 | 0 | 0 | 0 | 0 | 1 | 1 | 1 | 1 | 1 | 1 | P1245A |
| **21** | M | 78 | 28.24 | 8 | 10.03.2020 | 22.03.2020 | 1 | 1 | 2 | 1 | 0 | 0 | 0 | 0 | 1 | 1 | 0 | 0 | P1245A>C |
| **22** | M | 55 | 32.70 | 3 | 10.03.2020 | 20.03.2020 | 0 | 0 | 0 | 1 | 1 | 0 | 1 | 0 | 0 | 0 | 0 | 0 | P1245A>C |
| **23** | M | 85 | 27.40 | 4 | 11.03.2020 | 23.04.2020 | 0 | 0 | 0 | 1 | 1 | 0 | 1 | 1 | 0 | 1 | 1 | 0 | P1245A>C |
| **24** | F | 82 | 22.77 | 4 | 11.03.2020 | 19.03.2020 | 0 | 0 | 0 | 0 | 1 | 0 | 0 | 1 | 0 | 0 | 0 | 0 | P1245A |
| **25** | F | 78 | 30.49 | 3 | 11.03.2020 | 27.03.2020 | 0 | 0 | 0 | 0 | 0 | 0 | 0 | 0 | 0 | 1 | 0 | 0 | P1245C |
| **26** | F | 45 | 35.60 | 3 | 11.03.2020 | 16.03.2020 | 0 | 0 | 0 | 1 | 1 | 0 | 0 | 0 | 0 | 1 | 0 | 1 | P1245A>C |
| **27** | F | 72 | 18.15 | 4 | 11.03.2020 | 31.03.2020 | 0 | 0 | 0 | 1 | 1 | 0 | 0 | 0 | 0 | 0 | 0 | 0 | P1245A |
| **28** | M | 43 | NA | 4 | 11.03.2020 | 21.03.2020 | 0 | 1 | 1 | 1 | 1 | 0 | 1 | 0 | 0 | 0 | 0 | 0 | P1245A |
| **29** | F | 89 | 31.56 | 4 | 11.03.2020 | 07.04.2020 | 0 | 0 | 0 | 0 | 1 | 0 | 1 | 1 | 1 | 1 | 0 | 1 | P1245A |
| **30** | M | 74 | 27.56 | 8 | 11.03.2020 | 28.03.2020 | 1 | 0 | 0 | 0 | 1 | 0 | 0 | 1 | 0 | 0 | 0 | 0 | P1245A>C |
| **31** | F | 57 | 37.81 | 4 | 11.03.2020 | 28.03.2020 | 0 | 0 | 0 | 1 | 1 | 0 | 1 | 0 | 0 | 0 | 0 | 0 | P1245A>C |
| **32** | M | 50 | 21.62 | 3 | 11.03.2020 | 27.03.2020 | 0 | 0 | 0 | 0 | 1 | 0 | 0 | 0 | 0 | 0 | 0 | 0 | P1245A>C |
| **33** | M | 87 | 21.96 | 8 | 12.03.2020 | 23.03.2020 | 1 | 0 | 0 | 0 | 1 | 0 | 1 | 0 | 1 | 0 | 0 | 0 | P1245C |
| **34** | F | 78 | 28.00 | 3 | 12.03.2020 | 14.04.2020 | 0 | 0 | 0 | 0 | 0 | 0 | 0 | 0 | 1 | 1 | 0 | 1 | P1245A |
| **35** | F | 77 | NA | 4 | 12.03.2020 | 27.03.2020 | 0 | 0 | 0 | 1 | 1 | 1 | 1 | 0 | 0 | 0 | 0 | 0 | P1245A |
| **36** | M | 56 | NA | 8 | 12.03.2020 | 15.04.2020 | 1 | 1 | 34 | 1 | 1 | 0 | 0 | 0 | 0 | 1 | 0 | 0 | P1245A>C |
| **37** | F | 75 | 21.70 | 4 | 12.03.2020 | 30.04.2020 | 0 | 0 | 0 | 1 | 1 | 0 | 1 | 0 | 0 | 1 | 0 | 0 | P1245A |
| **38** | F | 77 | 22.86 | 4 | 13.03.2020 | 21.03.2020 | 0 | 0 | 0 | 0 | 1 | 0 | 1 | 0 | 1 | 1 | 0 | 0 | P1245A>C |
| **39** | F | 39 | 30.49 | 7 | 13.03.2020 | 23.04.2020 | 0 | 1 | 27 | 1 | 1 | 0 | 1 | 0 | 0 | 0 | 0 | 0 | P1245A |
| **40** | F | 73 | 23.56 | 3 | 13.03.2020 | 20.03.2020 | 0 | 0 | 0 | 1 | 1 | 0 | 0 | 0 | 0 | 1 | 0 | 0 | P1245A |
| **41** | M | 46 | NA | 4 | 13.03.2020 | 11.05.2020 | 0 | 1 | 1 | 1 | 1 | 0 | 1 | 0 | 0 | 0 | 0 | 0 | P1245A |
| **42** | F | 67 | NA | 3 | 13.03.2020 | 22.03.2020 | 0 | 0 | 0 | 1 | 1 | 0 | 1 | 0 | 0 | 0 | 1 | 0 | P1245A>C |
| **43** | M | 60 | 28.09 | 6 | 13.03.2020 | 17.04.2020 | 0 | 1 | 33 | 1 | 1 | 0 | 1 | 0 | 0 | 0 | 0 | 0 | P1245C |
| **44** | F | 54 | 36.71 | 4 | 13.03.2020 | 08.04.2020 | 0 | 0 | 0 | 1 | 1 | 0 | 1 | 0 | 0 | 0 | 0 | 0 | P1245C |
| **45** | F | 75 | 24.54 | 4 | 13.03.2020 | 01.04.2020 | 0 | 0 | 0 | 1 | 1 | 0 | 1 | 1 | 1 | 1 | 0 | 0 | P1245A>C |
| **46** | M | 80 | 29.31 | 5 | 13.03.2020 | 31.03.2020 | 0 | 0 | 0 | 1 | 1 | 0 | 1 | 0 | 0 | 0 | 0 | 0 | P1245A>C |
| **47** | F | 80 | 29.89 | 8 | 01.03.2020 | 20.03.2020 | 1 | 0 | 0 | 0 | 1 | 0 | 1 | 1 | 1 | 1 | 1 | 0 | P1245A>C |
| **48** | M | 88 | 30.37 | 3 | 13.03.2020 | 24.03.2020 | 0 | 0 | 0 | 0 | 1 | 0 | 1 | 1 | 1 | 1 | 0 | 0 | P1245C |
| **49** | M | 40 | NA | 3 | 13.03.2020 | 21.03.2020 | 0 | 0 | 0 | 1 | 1 | 0 | 1 | 0 | 0 | 0 | 0 | 0 | P1245A |
| **50** | M | 86 | NA | 4 | 13.03.2020 | 27.03.2020 | 0 | 0 | 0 | 1 | 1 | 0 | 1 | 0 | 1 | 0 | 0 | 0 | P1245A |
| **51** | M | 85 | 25.56 | 8 | 14.03.2020 | 02.04.2020 | 1 | 0 | 0 | 1 | 0 | 0 | 1 | 0 | 0 | 1 | 0 | 0 | P1245A |
| **52** | M | 77 | 30.11 | 8 | 14.03.2020 | 20.03.2020 | 1 | 0 | 0 | 1 | 1 | 1 | 1 | 1 | 1 | 1 | 0 | 0 | P1245A>C |
| **53** | F | 72 | 25.14 | 8 | 14.03.2020 | 21.03.2020 | 1 | 0 | 0 | 1 | 0 | 0 | 1 | 0 | 1 | 1 | 0 | 0 | P1245A |
| **54** | M | 87 | 25.70 | 4 | 14.03.2020 | 28.04.2020 | 0 | 0 | 0 | 1 | 1 | 0 | 1 | 0 | 0 | 1 | 0 | 0 | P1245A |
| **55** | F | 86 | NA | 4 | 14.03.2020 | 30.03.2020 | 0 | 0 | 0 | 0 | 1 | 1 | 1 | 1 | 1 | 1 | 0 | 0 | P1245A>C |
| **56** | F | 85 | 27.90 | 4 | 14.03.2020 | 01.04.2020 | 0 | 0 | 0 | 0 | 0 | 1 | 1 | 1 | 1 | 1 | 0 | 0 | P1245C |
| **57** | F | 51 | 41.50 | 5 | 14.03.2020 | 13.04.2020 | 0 | 0 | 0 | 0 | 1 | 0 | 0 | 0 | 1 | 1 | 0 | 0 | P1245A |
| **58** | F | 75 | 27.36 | 4 | 14.03.2020 | 27.03.2020 | 0 | 0 | 0 | 0 | 1 | 0 | 0 | 0 | 0 | 1 | 0 | 0 | P1245A>C |
| **59** | M | 81 | 26.00 | 8 | 15.03.2020 | 09.04.2020 | 1 | 1 | 35 | 1 | 1 | 1 | 0 | 1 | 0 | 1 | 0 | 0 | P1245C |
| **60** | M | 93 | 23.91 | 4 | 12.03.2020 | 10.05.2020 | 0 | 0 | 0 | 0 | 1 | 0 | 1 | 0 | 1 | 0 | 0 | 0 | P1245A>C |
| **61** | F | 77 | NA | 4 | 15.03.2020 | 31.03.2020 | 0 | 0 | 0 | 1 | 1 | 1 | 0 | 1 | 0 | 1 | 0 | 0 | P1245A |
| **62** | M | 49 | 26.74 | 3 | 15.03.2020 | 16.04.2020 | 0 | 0 | 0 | 1 | 1 | 0 | 0 | 0 | 0 | 0 | 0 | 0 | P1245A |
| **63** | M | 86 | 19.84 | 8 | 15.03.2020 | 24.03.2020 | 1 | 0 | 0 | 1 | 1 | 0 | 0 | 0 | 1 | 0 | 1 | 1 | P1245A |
| **64** | F | 80 | 30.80 | 4 | 15.03.2020 | 29.04.2020 | 0 | 0 | 0 | 1 | 1 | 0 | 1 | 0 | 1 | 1 | 0 | 0 | P1245A>C |
| **65** | M | 72 | 28.90 | 3 | 16.03.2020 | 19.03.2020 | 0 | 0 | 0 | 0 | 0 | 1 | 1 | 1 | 1 | 0 | 0 | 0 | P1245A>C |
| **66** | F | 79 | 22.32 | 4 | 09.03.2020 | 27.03.2020 | 0 | 0 | 0 | 1 | 1 | 0 | 0 | 0 | 0 | 0 | 0 | 1 | P1245A>C |
| **67** | M | 77 | 29.76 | 4 | 16.03.2020 | 28.04.2020 | 0 | 0 | 0 | 1 | 1 | 0 | 1 | 0 | 0 | 0 | 0 | 1 | P1245A>C |
| **68** | M | 66 | 20.71 | 4 | 16.03.2020 | 12.04.2020 | 0 | 0 | 0 | 1 | 1 | 0 | 1 | 0 | 1 | 1 | 0 | 0 | P1245A>C |
| **69** | F | 75 | 30.12 | 8 | 16.03.2020 | 22.03.2020 | 1 | 0 | 0 | 0 | 1 | 1 | 1 | 1 | 0 | 1 | 0 | 1 | P1245A>C |
| **70** | M | 67 | 30.53 | 6 | 16.03.2020 | 16.04.2020 | 0 | 1 | 22 | 1 | 1 | 0 | 1 | 0 | 1 | 1 | 0 | 0 | P1245A |
| **71** | M | 52 | 24.38 | 4 | 16.03.2020 | 20.03.2020 | 0 | 0 | 0 | 1 | 1 | 0 | 1 | 0 | 0 | 1 | 0 | 0 | P1245A |
| **72** | F | 65 | 22.76 | 3 | 16.03.2020 | 18.03.2020 | 0 | 0 | 0 | 1 | 0 | 0 | 0 | 0 | 0 | 1 | 0 | 0 | P1245C |
| **73** | M | 63 | 31.31 | 3 | 16.03.2020 | 11.05.2020 | 0 | 0 | 0 | 1 | 1 | 0 | 0 | 0 | 0 | 1 | 0 | 0 | P1245C |
| **74** | M | 79 | 23.93 | 8 | 16.03.2020 | 21.03.2020 | 1 | 1 | 1 | 1 | 1 | 0 | 1 | 0 | 1 | 1 | 0 | 0 | P1245A>C |
| **75** | F | 53 | 27.31 | 3 | 17.03.2020 | 20.03.2020 | 0 | 0 | 0 | 1 | 1 | 0 | 0 | 0 | 0 | 0 | 0 | 0 | P1245A>C |
| **76** | M | 73 | 24.69 | 4 | 16.03.2020 | 30.03.2020 | 0 | 0 | 0 | 1 | 1 | 0 | 0 | 0 | 0 | 1 | 0 | 0 | P1245A>C |
| **77** | F | 86 | 21.87 | 4 | 17.03.2020 | 27.03.2020 | 0 | 0 | 0 | 1 | 0 | 0 | 0 | 0 | 1 | 1 | 0 | 0 | P1245A |
| **78** | M | 81 | 24.26 | 8 | 17.03.2020 | 23.03.2020 | 1 | 0 | 0 | 1 | 1 | 0 | 1 | 0 | 0 | 1 | 0 | 0 | P1245A>C |
| **79** | M | 65 | 25.99 | 5 | 17.03.2020 | 24.03.2020 | 0 | 0 | 0 | 0 | 1 | 0 | 1 | 0 | 0 | 1 | 0 | 0 | P1245A>C |
| **80** | M | 53 | 31.66 | 4 | 17.03.2020 | 24.03.2020 | 0 | 0 | 0 | 1 | 1 | 0 | 1 | 0 | 0 | 0 | 0 | 0 | P1245A>C |
| **81** | M | 80 | 30.12 | 3 | 17.03.2020 | 27.03.2020 | 0 | 0 | 0 | 1 | 0 | 1 | 1 | 0 | 1 | 1 | 0 | 0 | P1245A>C |
| **82** | M | 29 | 27.15 | 1 | 18.03.2020 | 30.04.2020 | 0 | 0 | 0 | 1 | 1 | 0 | 1 | 0 | 0 | 1 | 0 | 0 | P1245A |
| **83** | F | 81 | 37.06 | 4 | 18.03.2020 | 01.04.2020 | 0 | 0 | 0 | 1 | 1 | 0 | 1 | 1 | 1 | 1 | 0 | 0 | P1245A |
| **84** | M | 77 | 30.00 | 4 | 18.03.2020 | 27.03.2020 | 0 | 1 | 2 | 1 | 1 | 0 | 0 | 0 | 1 | 1 | 1 | 0 | P1245C |
| **85** | F | 75 | 30.01 | 3 | 18.03.2020 | 26.03.2020 | 0 | 0 | 0 | 1 | 1 | 1 | 1 | 0 | 1 | 1 | 0 | 0 | P1245A>C |
| **86** | M | 65 | 24.53 | 3 | 18.03.2020 | 25.03.2020 | 0 | 0 | 0 | 1 | 0 | 0 | 1 | 0 | 0 | 0 | 0 | 0 | P1245A>C |
| **87** | F | 58 | 28.20 | 4 | 18.03.2020 | 27.03.2020 | 0 | 0 | 0 | 0 | 1 | 0 | 1 | 0 | 0 | 0 | 0 | 0 | P1245C |
| **88** | F | 55 | 18.98 | 4 | 18.03.2020 | 24.03.2020 | 0 | 0 | 0 | 1 | 1 | 0 | 0 | 0 | 0 | 0 | 0 | 0 | P1245A |
| **89** | M | 72 | 23.95 | 4 | 18.03.2020 | 31.03.2020 | 0 | 0 | 0 | 0 | 0 | 0 | 0 | 0 | 0 | 1 | 0 | 1 | P1245A>C |
| **90** | M | 77 | 35.00 | 8 | 18.03.2020 | 29.03.2020 | 1 | 0 | 0 | 1 | 1 | 0 | 0 | 0 | 0 | 0 | 0 | 0 | P1245A |
| **91** | M | 57 | 24.16 | 4 | 18.03.2020 | 03.04.2020 | 0 | 0 | 0 | 1 | 1 | 0 | 1 | 0 | 0 | 0 | 0 | 0 | P1245C |
| **92** | M | 69 | 27.09 | 4 | 18.03.2020 | 16.04.2020 | 0 | 0 | 0 | 1 | 1 | 0 | 1 | 1 | 0 | 1 | 1 | 0 | P1245A |
| **93** | M | 66 | 26.06 | 4 | 18.03.2020 | 03.04.2020 | 0 | 1 | 1 | 1 | 1 | 1 | 1 | 0 | 0 | 1 | 0 | 0 | P1245A |
| **94** | M | 83 | 118.98 | 8 | 18.03.2020 | 30.03.2020 | 1 | 0 | 0 | 0 | 1 | 0 | 1 | 0 | 1 | 1 | 0 | 1 | P1245A |
| **95** | F | 59 | 25.72 | 6 | 19.03.2020 | 30.04.2020 | 0 | 1 | 1 | 1 | 1 | 0 | 1 | 0 | 0 | 0 | 0 | 0 | P1245A |
| **96** | M | 82 | NA | 8 | 19.03.2020 | 25.03.2020 | 1 | 0 | 0 | 0 | 1 | 1 | 0 | 0 | 1 | 1 | 0 | 0 | P1245A |
| **97** | M | 58 | 26.52 | 8 | 19.03.2020 | 01.04.2020 | 0 | 1 | 48 | 1 | 1 | 0 | 1 | 0 | 1 | 0 | 0 | 0 | P1245A>C |
| **98** | F | 38 | 28.70 | 6 | 19.03.2020 | 02.04.2020 | 0 | 1 | 1 | 0 | 1 | 0 | 1 | 0 | 0 | 0 | 0 | 0 | P1245A>C |
| **99** | M | 60 | 30.49 | 4 | 19.03.2020 | 16.04.2020 | 0 | 0 | 0 | 1 | 1 | 1 | 1 | 0 | 0 | 0 | 0 | 0 | P1245A |
| **100** | M | 58 | 26.95 | 7 | 19.03.2020 | 15.04.2020 | 0 | 1 | 51 | 1 | 1 | 0 | 1 | 1 | 0 | 1 | 0 | 0 | P1245A>C |
| **101** | M | 69 | 25.19 | 4 | 19.03.2020 | 30.03.2020 | 0 | 0 | 0 | 1 | 1 | 0 | 0 | 0 | 0 | 1 | 0 | 0 | P1245A |
| **102** | M | 60 | 20.22 | 6 | 19.03.2020 | 01.04.2020 | 0 | 1 | 1 | 1 | 0 | 1 | 1 | 0 | 0 | 0 | 0 | 0 | P1245A |
| **103** | M | 58 | 27.55 | 4 | 20.03.2020 | 29.03.2020 | 0 | 0 | 0 | 1 | 1 | 0 | 0 | 1 | 1 | 1 | 1 | 0 | P1245A>C |
| **104** | F | 74 | 23.14 | 5 | 19.03.2020 | 06.05.2020 | 0 | 0 | 0 | 1 | 1 | 0 | 0 | 0 | 0 | 0 | 1 | 0 | P1245A |
| **105** | F | 59 | 27.44 | 4 | 20.03.2020 | 28.03.2020 | 0 | 0 | 0 | 1 | 0 | 0 | 0 | 0 | 0 | 1 | 0 | 0 | P1245A |
| **106** | F | 75 | 28.00 | 8 | 20.03.2020 | 22.03.2020 | 1 | 1 | 1 | 0 | 0 | 1 | 1 | 1 | 1 | 1 | 0 | 0 | P1245A>C |
| **107** | M | 52 | 30.78 | 6 | 20.03.2020 | 01.04.2020 | 0 | 1 | 40 | 1 | 1 | 0 | 1 | 0 | 0 | 1 | 0 | 0 | P1245A>C |
| **108** | M | 76 | 23.68 | 8 | 20.03.2020 | 31.03.2020 | 1 | 0 | 0 | 0 | 1 | 1 | 1 | 1 | 1 | 1 | 1 | 1 | P1245A>C |
| **109** | F | 90 | 34.26 | 4 | 20.03.2020 | 16.04.2020 | 0 | 0 | 0 | 1 | 1 | 0 | 0 | 0 | 1 | 1 | 1 | 0 | P1245A |
| **110** | F | 70 | 29.10 | 5 | 20.03.2020 | 06.04.2020 | 0 | 0 | 0 | 1 | 1 | 1 | 0 | 0 | 0 | 1 | 0 | 0 | P1245A |
| **111** | F | 66 | 26.12 | 4 | 20.03.2020 | 27.03.2020 | 0 | 0 | 0 | 1 | 0 | 0 | 1 | 0 | 1 | 0 | 0 | 0 | P1245A>C |
| **112** | F | 88 | 25.90 | 8 | 20.03.2020 | 27.03.2020 | 1 | 0 | 0 | 1 | 1 | 0 | 1 | 0 | 0 | 1 | 1 | 0 | P1245A |
| **113** | M | 58 | 24.70 | 6 | 20.03.2020 | 19.04.2020 | 0 | 1 | 6 | 1 | 1 | 0 | 1 | 0 | 0 | 1 | 0 | 0 | P1245A>C |
| **114** | F | 77 | NA | 4 | 20.03.2020 | 06.04.2020 | 0 | 0 | 0 | 0 | 1 | 1 | 0 | 0 | 0 | 1 | 0 | 0 | P1245C |
| **115** | M | 61 | 40.88 | 4 | 20.03.2020 | 27.03.2020 | 0 | 0 | 0 | 1 | 1 | 0 | 1 | 1 | 0 | 1 | 0 | 0 | P1245A>C |
| **116** | M | 67 | 28.10 | 5 | 20.03.2020 | 28.03.2020 | 0 | 0 | 0 | 1 | 1 | 0 | 1 | 0 | 0 | 0 | 0 | 0 | P1245A>C |
| **117** | F | 70 | 22.71 | 4 | 20.03.2020 | 16.04.2020 | 0 | 0 | 0 | 1 | 1 | 0 | 1 | 0 | 0 | 0 | 1 | 1 | P1245A>C |
| **118** | M | 74 | 23.82 | 4 | 20.03.2020 | 18.04.2020 | 0 | 0 | 0 | 0 | 1 | 1 | 1 | 0 | 1 | 1 | 0 | 0 | P1245A>C |
| **119** | F | 80 | 48.44 | 8 | 21.03.2020 | 26.03.2020 | 1 | 0 | 0 | 1 | 1 | 1 | 0 | 1 | 0 | 0 | 1 | 1 | P1245A |
| **120** | F | 71 | 22.10 | 8 | 21.03.2020 | 22.03.2020 | 1 | 1 | 1 | 1 | 0 | 0 | 0 | 1 | 1 | 1 | 0 | 1 | P1245A |
| **121** | M | 66 | 29.40 | 8 | 21.03.2020 | 14.04.2020 | 1 | 1 | 22 | 1 | 1 | 0 | 1 | 0 | 1 | 0 | 1 | 0 | P1245A |
| **122** | M | 72 | 24.67 | 4 | 21.03.2020 | 27.03.2020 | 0 | 0 | 0 | 0 | 1 | 0 | 0 | 0 | 1 | 0 | 1 | 0 | P1245A |
| **123** | F | 86 | 22.47 | 4 | 22.03.2020 | 15.04.2020 | 0 | 0 | 0 | 1 | 1 | 1 | 1 | 1 | 0 | 1 | 0 | 0 | P1245A>C |
| **124** | M | 79 | 27.41 | 4 | 22.03.2020 | 17.04.2020 | 0 | 0 | 0 | 1 | 0 | 0 | 0 | 0 | 1 | 1 | 0 | 0 | P1245A |
| **125** | M | 61 | 24.11 | 4 | 22.03.2020 | 08.04.2020 | 0 | 0 | 0 | 1 | 1 | 1 | 1 | 0 | 0 | 0 | 0 | 0 | P1245A>C |
| **126** | F | 82 | NA | 3 | 22.03.2020 | 01.04.2020 | 0 | 0 | 0 | 1 | 1 | 1 | 1 | 0 | 0 | 0 | 0 | 0 | P1245A>C |
| **127** | M | 69 | 17.94 | 3 | 23.03.2020 | 24.04.2020 | 0 | 0 | 0 | 1 | 1 | 0 | 0 | 1 | 0 | 1 | 1 | 1 | P1245A |
| **128** | M | 76 | 28.19 | 3 | 23.03.2020 | 30.03.2020 | 0 | 0 | 0 | 0 | 1 | 0 | 1 | 0 | 1 | 1 | 1 | 1 | P1245A>C |
| **129** | M | 77 | 30.10 | 6 | 23.03.2020 | 01.04.2020 | 0 | 1 | 48 | 1 | 1 | 0 | 1 | 0 | 0 | 1 | 0 | 1 | P1245A>C |
| **130** | F | 79 | 33.28 | 8 | 23.03.2020 | 04.05.2020 | 1 | 1 | 5 | 1 | 1 | 0 | 1 | 1 | 1 | 1 | 1 | 1 | P1245A |
| **131** | M | 84 | 25.20 | 4 | 23.03.2020 | 09.04.2020 | 0 | 0 | 0 | 1 | 1 | 0 | 1 | 0 | 0 | 1 | 0 | 0 | P1245C |
| **132** | F | 83 | NA | 4 | 23.03.2020 | 31.03.2020 | 0 | 0 | 0 | 0 | 1 | 1 | 1 | 0 | 0 | 1 | 0 | 0 | P1245A |
| **133** | M | 86 | 22.85 | 3 | 23.03.2020 | 02.04.2020 | 0 | 0 | 0 | 1 | 1 | 0 | 0 | 0 | 1 | 1 | 0 | 1 | P1245A>C |
| **134** | F | 88 | NA | 8 | 23.03.2020 | 30.03.2020 | 1 | 0 | 0 | 1 | 1 | 0 | 1 | 0 | 0 | 1 | 0 | 0 | P1245C |
| **135** | M | 98 | 20.80 | 8 | 23.03.2020 | 01.04.2020 | 1 | 0 | 0 | 1 | 1 | 0 | 1 | 0 | 0 | 0 | 0 | 0 | P1245A |
| **136** | M | 72 | 31.07 | 6 | 23.03.2020 | 28.03.2020 | 0 | 1 | 3 | 1 | 1 | 0 | 1 | 0 | 0 | 1 | 0 | 0 | P1245A>C |
| **137** | M | 51 | 27.30 | 4 | 23.03.2020 | 11.04.2020 | 0 | 0 | 0 | 1 | 1 | 0 | 1 | 0 | 0 | 0 | 0 | 0 | P1245A>C |
| **138** | M | 70 | 28.60 | 4 | 24.03.2020 | 15.04.2020 | 0 | 0 | 0 | 1 | 1 | 0 | 1 | 0 | 0 | 0 | 1 | 0 | P1245A>C |
| **139** | M | 52 | 24.30 | 4 | 23.03.2020 | 01.04.2020 | 0 | 0 | 0 | 1 | 1 | 0 | 1 | 0 | 0 | 0 | 1 | 0 | P1245C |
| **140** | M | 77 | 20.30 | 4 | 24.03.2020 | 06.04.2020 | 0 | 0 | 0 | 1 | 0 | 0 | 0 | 0 | 1 | 0 | 0 | 0 | P1245A>C |
| **141** | F | 69 | 26.50 | 3 | 24.03.2020 | 07.04.2020 | 0 | 0 | 0 | 1 | 1 | 0 | 1 | 0 | 0 | 0 | 0 | 0 | P1245A>C |
| **142** | M | 80 | 31.07 | 8 | 12.03.2020 | 26.03.2020 | 1 | 0 | 0 | 1 | 1 | 0 | 1 | 1 | 1 | 0 | 1 | 1 | P1245A>C |
| **143** | F | 81 | 22.38 | 8 | 24.03.2020 | 06.04.2020 | 1 | 0 | 0 | 1 | 0 | 0 | 0 | 1 | 1 | 1 | 0 | 0 | P1245C |
| **144** | M | 70 | 39.63 | 8 | 24.03.2020 | 16.04.2020 | 1 | 1 | 24 | 1 | 1 | 0 | 1 | 1 | 1 | 1 | 0 | 0 | P1245C |
| **145** | F | 75 | 27.78 | 4 | 24.03.2020 | 09.04.2020 | 0 | 0 | 0 | 1 | 0 | 1 | 1 | 1 | 0 | 1 | 1 | 0 | P1245A>C |
| **146** | M | 86 | 27.20 | 4 | 24.03.2020 | 24.04.2020 | 0 | 0 | 0 | 1 | 1 | 0 | 0 | 1 | 1 | 0 | 0 | 0 | P1245A |
| **147** | F | 64 | 36.10 | 4 | 24.03.2020 | 25.03.2020 | 0 | 0 | 0 | 1 | 1 | 0 | 0 | 1 | 0 | 1 | 0 | 0 | P1245A>C |
| **148** | F | 89 | 29.30 | 4 | 24.03.2020 | 02.04.2020 | 0 | 0 | 0 | 0 | 0 | 1 | 0 | 1 | 1 | 1 | 0 | 0 | P1245C |
| **149** | F | 77 | 33.10 | 5 | 24.03.2020 | 03.04.2020 | 0 | 0 | 0 | 1 | 0 | 1 | 0 | 1 | 0 | 0 | 0 | 0 | P1245A |
| **150** | F | 53 | 34.40 | 4 | 24.03.2020 | 22.04.2020 | 0 | 0 | 0 | 1 | 1 | 0 | 0 | 0 | 0 | 0 | 0 | 0 | P1245A>C |
| **151** | M | 74 | 29.20 | 4 | 25.03.2020 | 14.04.2020 | 0 | 0 | 0 | 1 | 1 | 1 | 0 | 1 | 1 | 1 | 0 | 0 | P1245A |
| **152** | M | 87 | 19.30 | 3 | 25.03.2020 | 29.03.2020 | 1 | 0 | 0 | 1 | 0 | 0 | 0 | 0 | 1 | 0 | 0 | 1 | P1245A>C |
| **153** | M | 78 | 34.61 | 8 | 25.03.2020 | 31.03.2020 | 1 | 1 | 2 | 1 | 0 | 0 | 1 | 1 | 1 | 1 | 0 | 1 | P1245A |
| **154** | F | 79 | 23.55 | 3 | 25.03.2020 | 15.04.2020 | 0 | 0 | 0 | 1 | 1 | 0 | 0 | 0 | 0 | 1 | 0 | 0 | P1245A |
| **155** | M | 48 | 30.30 | 4 | 25.03.2020 | 26.03.2020 | 0 | 0 | 0 | 1 | 0 | 0 | 1 | 0 | 0 | 0 | 0 | 0 | P1245A>C |
| **156** | M | 58 | 33.46 | 4 | 25.03.2020 | 06.04.2020 | 0 | 1 | 1 | 1 | 1 | 0 | 1 | 0 | 0 | 1 | 0 | 0 | P1245A |
| **157** | M | 56 | 30.53 | 4 | 25.03.2020 | 05.04.2020 | 0 | 0 | 0 | 1 | 0 | 0 | 0 | 0 | 0 | 1 | 0 | 0 | P1245A>C |
| **158** | F | 55 | 25.88 | 4 | 25.03.2020 | 01.04.2020 | 0 | 0 | 0 | 1 | 0 | 0 | 0 | 0 | 0 | 0 | 0 | 0 | P1245A>C |
| **159** | F | 85 | 25.16 | 8 | 25.03.2020 | 05.04.2020 | 1 | 0 | 0 | 1 | 1 | 0 | 0 | 1 | 1 | 1 | 0 | 0 | P1245A>C |
| **160** | M | 64 | 27.68 | 4 | 25.03.2020 | 15.04.2020 | 0 | 0 | 0 | 1 | 1 | 0 | 1 | 0 | 1 | 0 | 1 | 1 | P1245A>C |
| **161** | F | 68 | 21.29 | 6 | 25.03.2020 | 08.05.2020 | 0 | 1 | 9 | 1 | 1 | 0 | 1 | 0 | 0 | 0 | 0 | 1 | P1245A>C |
| **162** | M | 63 | 29.30 | 8 | 26.03.2020 | 03.04.2020 | 1 | 0 | 0 | 1 | 1 | 0 | 1 | 0 | 0 | 1 | 0 | 0 | P1245A>C |
| **163** | M | 84 | 23.58 | 3 | 26.03.2020 | 08.05.2020 | 0 | 0 | 0 | 1 | 1 | 0 | 1 | 0 | 1 | 1 | 0 | 0 | P1245C |
| **164** | F | 58 | 47.39 | 1 | 26.03.2020 | 08.04.2020 | 0 | 0 | 0 | 0 | 0 | 0 | 1 | 0 | 0 | 0 | 0 | 1 | P1245A>C |
| **165** | F | 54 | 21.74 | 3 | 26.03.2020 | 31.03.2020 | 0 | 0 | 0 | 1 | 1 | 0 | 1 | 0 | 0 | 0 | 0 | 0 | P1245A>C |
| **166** | F | 80 | 26.90 | 4 | 26.03.2020 | 10.04.2020 | 0 | 0 | 0 | 0 | 1 | 1 | 0 | 0 | 1 | 0 | 0 | 0 | P1245A |
| **167** | M | 78 | 28.83 | 4 | 26.03.2020 | 27.03.2020 | 0 | 0 | 0 | 1 | 1 | 0 | 1 | 0 | 0 | 1 | 1 | 0 | P1245C |
| **168** | F | 75 | 29.80 | 3 | 26.03.2020 | 30.03.2020 | 0 | 0 | 0 | 1 | 0 | 0 | 0 | 0 | 1 | 1 | 1 | 0 | P1245C |
| **169** | M | 67 | 22.14 | 8 | 26.03.2020 | 27.04.2020 | 1 | 1 | 31 | 1 | 1 | 0 | 1 | 0 | 0 | 0 | 1 | 0 | P1245A>C |
| **170** | M | 70 | 25.71 | 4 | 26.03.2020 | 20.04.2020 | 0 | 0 | 0 | 1 | 1 | 1 | 0 | 0 | 0 | 0 | 0 | 0 | P1245A |
| **171** | M | 56 | NA | 3 | 26.03.2020 | 27.03.2020 | 0 | 0 | 0 | 1 | 1 | 0 | 1 | 0 | 0 | 1 | 0 | 0 | P1245A>C |
| **172** | M | 80 | NA | 3 | 26.03.2020 | 27.03.2020 | 0 | 0 | 0 | 0 | 0 | 0 | 0 | 0 | 0 | 0 | 0 | 0 | P1245A>C |
| **173** | F | 45 | 24.57 | 4 | 27.03.2020 | 04.04.2020 | 0 | 0 | 0 | 1 | 1 | 0 | 0 | 0 | 0 | 0 | 0 | 0 | P1245C |
| **174** | M | 73 | NA | 4 | 27.03.2020 | 27.03.2020 | 0 | 0 | 0 | 1 | 1 | 0 | 0 | 1 | 0 | 0 | 0 | 0 | P1245A |
| **175** | F | 69 | 31.20 | 3 | 27.03.2020 | 28.03.2020 | 0 | 0 | 0 | 0 | 1 | 0 | 0 | 1 | 1 | 0 | 0 | 0 | P1245A>C |
| **176** | F | 68 | 31.64 | 4 | 27.03.2020 | 15.04.2020 | 0 | 0 | 0 | 1 | 0 | 0 | 0 | 0 | 0 | 0 | 0 | 0 | P1245A>C |
| **177** | M | 55 | 26.07 | 4 | 27.03.2020 | 30.03.2020 | 0 | 0 | 0 | 1 | 0 | 1 | 0 | 0 | 0 | 0 | 0 | 0 | P1245A |
| **178** | F | 70 | 32.70 | 4 | 27.03.2020 | 29.03.2020 | 0 | 0 | 0 | 1 | 1 | 0 | 0 | 0 | 0 | 0 | 0 | 0 | P1245A>C |
| **179** | M | 76 | 24.58 | 4 | 27.03.2020 | 10.04.2020 | 0 | 0 | 0 | 1 | 1 | 0 | 1 | 0 | 1 | 0 | 0 | 0 | P1245A>C |
| **180** | M | 63 | 29.42 | 4 | 27.03.2020 | 29.04.2020 | 0 | 0 | 0 | 1 | 1 | 1 | 0 | 0 | 0 | 0 | 0 | 0 | P1245C |
| **181** | F | 68 | 30.90 | 4 | 27.03.2020 | 27.03.2020 | 0 | 0 | 0 | 1 | 1 | 1 | 1 | 0 | 0 | 1 | 0 | 0 | P1245C |
| **182** | M | 75 | 32.10 | 4 | 27.03.2020 | 01.04.2020 | 0 | 0 | 0 | 1 | 0 | 1 | 0 | 1 | 1 | 1 | 0 | 0 | P1245A>C |
| **183** | M | 53 | 29.10 | 4 | 26.03.2020 | 01.04.2020 | 0 | 0 | 0 | 0 | 1 | 0 | 1 | 0 | 0 | 0 | 0 | 0 | P1245A |
| **184** | M | 67 | 32.18 | 3 | 27.03.2020 | 28.03.2020 | 0 | 0 | 0 | 0 | 1 | 1 | 0 | 0 | 1 | 0 | 0 | 1 | P1245A>C |
| **185** | M | 62 | 28.65 | 5 | 27.03.2020 | 28.03.2020 | 0 | 0 | 0 | 1 | 1 | 0 | 1 | 0 | 1 | 0 | 0 | 0 | P1245A>C |
| **186** | M | 69 | 22.80 | 4 | 27.03.2020 | 08.04.2020 | 0 | 0 | 0 | 1 | 1 | 0 | 1 | 1 | 1 | 1 | 1 | 0 | P1245A |
| **187** | M | 58 | NA | 5 | 27.03.2020 | 01.04.2020 | 0 | 1 | 1 | 1 | 0 | 0 | 1 | 0 | 0 | 0 | 1 | 0 | P1245A |
| **188** | F | 67 | 28.30 | 8 | 27.03.2020 | 04.04.2020 | 1 | 0 | 0 | 0 | 1 | 1 | 0 | 0 | 1 | 1 | 0 | 0 | P1245A>C |
| **189** | M | 77 | 26.22 | 5 | 27.03.2020 | 03.04.2020 | 0 | 1 | 1 | 1 | 1 | 1 | 1 | 0 | 0 | 1 | 0 | 0 | P1245A>C |
| **190** | M | 61 | 25.30 | 4 | 26.03.2020 | 04.04.2020 | 0 | 0 | 0 | 1 | 1 | 0 | 1 | 0 | 0 | 0 | 0 | 0 | P1245C |
| **191** | M | 80 | NA | 8 | 27.03.2020 | 31.03.2020 | 1 | 0 | 0 | 1 | 1 | 0 | 1 | 0 | 0 | 0 | 0 | 0 | P1245A |
| **192** | F | 72 | 25.27 | 4 | 27.03.2020 | 16.04.2020 | 0 | 0 | 0 | 1 | 1 | 0 | 1 | 0 | 0 | 0 | 1 | 0 | P1245A |
| **193** | F | 87 | 27.83 | 4 | 27.03.2020 | 21.04.2020 | 0 | 0 | 0 | 1 | 1 | 1 | 1 | 1 | 0 | 1 | 0 | 1 | P1245A>C |
| **194** | M | 58 | 33.39 | 7 | 28.03.2020 | 08.04.2020 | 0 | 1 | 1 | 0 | 1 | 0 | 0 | 1 | 1 | 1 | 1 | 0 | P1245A |
| **195** | M | 81 | 26.64 | 4 | 27.03.2020 | 08.04.2020 | 0 | 0 | 0 | 1 | 1 | 0 | 0 | 0 | 0 | 0 | 0 | 0 | P1245A>C |
| **196** | M | 74 | 26.15 | 4 | 28.03.2020 | 03.04.2020 | 0 | 0 | 0 | 1 | 1 | 1 | 1 | 0 | 0 | 1 | 0 | 0 | P1245C |
| **197** | M | 60 | 22.90 | 6 | 28.03.2020 | 27.04.2020 | 0 | 1 | 21 | 0 | 1 | 0 | 0 | 0 | 0 | 0 | 0 | 0 | P1245A>C |
| **198** | F | 77 | 42.11 | 8 | 28.03.2020 | 11.04.2020 | 1 | 0 | 0 | 1 | 1 | 0 | 0 | 0 | 1 | 1 | 1 | 0 | P1245A>C |
| **199** | F | 68 | 24.40 | 3 | 28.03.2020 | 14.04.2020 | 0 | 0 | 0 | 0 | 0 | 0 | 1 | 0 | 0 | 0 | 0 | 0 | P1245A>C |
| **200** | M | 84 | 26.57 | 4 | 28.03.2020 | 17.04.2020 | 0 | 0 | 0 | 1 | 1 | 0 | 1 | 0 | 0 | 1 | 0 | 1 | P1245A |
| **201** | M | 82 | 24.30 | 4 | 28.03.2020 | 11.04.2020 | 0 | 0 | 0 | 0 | 1 | 0 | 0 | 0 | 0 | 0 | 0 | 0 | P1245A>C |
| **202** | F | 73 | 22.77 | 4 | 29.03.2020 | 29.03.2020 | 0 | 0 | 0 | 0 | 1 | 0 | 0 | 1 | 0 | 0 | 0 | 0 | P1245C |
| **203** | F | 68 | 34.51 | 6 | 29.03.2020 | 01.04.2020 | 0 | 1 | 31 | 1 | 1 | 1 | 1 | 1 | 0 | 0 | 0 | 0 | P1245A>C |
| **204** | M | 79 | 22.98 | 8 | 29.03.2020 | 29.03.2020 | 1 | 0 | 0 | 1 | 1 | 0 | 0 | 0 | 1 | 0 | 0 | 1 | P1245A>C |
| **205** | M | 78 | 24.30 | 4 | 29.03.2020 | 29.03.2020 | 0 | 0 | 0 | 1 | 1 | 0 | 1 | 0 | 0 | 0 | 0 | 0 | P1245A |
| **206** | F | 50 | 30.02 | 4 | 29.03.2020 | 07.04.2020 | 0 | 0 | 0 | 1 | 1 | 0 | 1 | 0 | 0 | 0 | 0 | 0 | P1245A |
| **207** | M | 75 | 30.89 | 6 | 29.03.2020 | 01.04.2020 | 0 | 1 | 38 | 1 | 1 | 1 | 1 | 1 | 1 | 1 | 0 | 0 | P1245A |
| **208** | F | 86 | 32.00 | 8 | 28.03.2020 | 01.04.2020 | 1 | 0 | 0 | 1 | 0 | 0 | 1 | 0 | 1 | 1 | 0 | 0 | P1245A>C |
| **209** | F | 76 | 31.20 | 4 | 22.03.2020 | 10.04.2020 | 0 | 0 | 0 | 1 | 1 | 0 | 1 | 1 | 0 | 0 | 0 | 0 | P1245A |
| **210** | F | 80 | 24.61 | 4 | 30.03.2020 | 14.04.2020 | 1 | 0 | 0 | 1 | 1 | 0 | 1 | 0 | 0 | 1 | 0 | 1 | P1245A>C |
| **211** | M | 81 | 27.30 | 4 | 30.03.2020 | 05.05.2020 | 0 | 0 | 0 | 0 | 1 | 0 | 0 | 0 | 0 | 1 | 1 | 1 | P1245A |
| **212** | F | 51 | 21.40 | 4 | 30.03.2020 | 06.04.2020 | 0 | 0 | 0 | 1 | 1 | 0 | 1 | 0 | 0 | 0 | 0 | 0 | P1245C |
| **213** | M | 76 | 29.55 | 3 | 30.03.2020 | 10.04.2020 | 0 | 0 | 0 | 1 | 1 | 0 | 0 | 1 | 1 | 1 | 0 | 0 | P1245A>C |
| **214** | M | 74 | 28.93 | 4 | 30.03.2020 | 14.04.2020 | 0 | 0 | 0 | 1 | 1 | 1 | 0 | 0 | 1 | 1 | 0 | 0 | P1245C |
| **215** | M | 84 | 29.21 | 3 | 30.03.2020 | 08.04.2020 | 0 | 0 | 0 | 1 | 1 | 1 | 0 | 0 | 1 | 1 | 0 | 0 | P1245A |
| **216** | F | 64 | 26.30 | 3 | 30.03.2020 | 31.03.2020 | 0 | 0 | 0 | 1 | 1 | 1 | 0 | 1 | 1 | 1 | 0 | 0 | P1245A |
| **217** | F | 76 | 42.82 | 5 | 30.03.2020 | 17.04.2020 | 0 | 0 | 0 | 1 | 0 | 1 | 1 | 1 | 0 | 0 | 0 | 0 | P1245A |
| **218** | F | 94 | 24.29 | 4 | 30.03.2020 | 10.04.2020 | 0 | 0 | 0 | 1 | 1 | 0 | 0 | 0 | 1 | 0 | 0 | 0 | P1245A>C |
| **219** | M | 68 | NA | 3 | 31.03.2020 | 31.03.2020 | 0 | 0 | 0 | 1 | 1 | 0 | 1 | 0 | 0 | 0 | 0 | 0 | P1245C |
| **220** | M | 76 | 39.86 | 4 | 31.03.2020 | 21.04.2020 | 0 | 0 | 0 | 1 | 1 | 0 | 0 | 1 | 1 | 1 | 1 | 0 | P1245A>C |
| **221** | F | 77 | 29.32 | 4 | 31.03.2020 | 20.04.2020 | 0 | 0 | 0 | 0 | 0 | 0 | 0 | 1 | 0 | 0 | 1 | 0 | P1245A>C |
| **222** | M | 64 | NA | 4 | 31.03.2020 | 21.04.2020 | 0 | 0 | 0 | 1 | 1 | 0 | 1 | 1 | 0 | 0 | 0 | 0 | P1245C |
| **223** | F | 83 | 29.75 | 4 | 31.03.2020 | 19.05.2020 | 0 | 1 | 1 | 1 | 0 | 1 | 1 | 1 | 1 | 1 | 0 | 0 | P1245A |
| **224** | M | 67 | 27.65 | 4 | 31.03.2020 | 12.04.2020 | 0 | 0 | 0 | 1 | 1 | 0 | 1 | 0 | 0 | 0 | 0 | 1 | P1245C |
| **225** | M | 71 | 27.76 | 4 | 31.03.2020 | 07.04.2020 | 0 | 0 | 0 | 1 | 0 | 0 | 1 | 0 | 1 | 1 | 1 | 0 | P1245A>C |
| **226** | F | 84 | 31.17 | 4 | 31.03.2020 | 15.04.2020 | 0 | 0 | 0 | 0 | 1 | 0 | 1 | 0 | 0 | 1 | 0 | 0 | P1245A |
| **227** | F | 81 | 24.86 | 4 | 01.04.2020 | 08.05.2020 | 0 | 0 | 0 | 1 | 0 | 0 | 0 | 0 | 1 | 1 | 0 | 0 | P1245A |
| **228** | M | 72 | 32.87 | 4 | 01.04.2020 | 07.04.2020 | 0 | 0 | 0 | 1 | 0 | 0 | 1 | 1 | 0 | 1 | 0 | 0 | P1245A>C |
| **229** | M | 43 | 24.98 | 4 | 01.04.2020 | 04.04.2020 | 0 | 0 | 0 | 0 | 1 | 0 | 0 | 0 | 0 | 0 | 0 | 0 | P1245A>C |
| **230** | F | 90 | 25.91 | 4 | 01.04.2020 | 09.04.2020 | 0 | 0 | 0 | 1 | 1 | 0 | 0 | 1 | 1 | 1 | 0 | 0 | P1245A |
| **231** | M | 81 | 15.00 | 8 | 01.04.2020 | 03.04.2020 | 1 | 0 | 0 | 0 | 0 | 0 | 1 | 0 | 1 | 0 | 0 | 0 | P1245A>C |
| **232** | M | 79 | 27.47 | 3 | 02.04.2020 | 20.04.2020 | 0 | 0 | 0 | 0 | 0 | 0 | 0 | 1 | 0 | 1 | 0 | 1 | P1245A>C |
| **233** | M | 70 | 28.09 | 4 | 02.04.2020 | 14.04.2020 | 0 | 0 | 0 | 1 | 1 | 0 | 1 | 0 | 0 | 0 | 0 | 0 | P1245A>C |
| **234** | F | 70 | 18.16 | 4 | 02.04.2020 | 06.04.2020 | 0 | 0 | 0 | 0 | 0 | 1 | 0 | 0 | 0 | 0 | 0 | 0 | P1245A |
| **235** | F | 62 | 28.48 | 4 | 01.04.2020 | 24.04.2020 | 0 | 0 | 0 | 0 | 1 | 0 | 1 | 0 | 0 | 0 | 0 | 1 | P1245C |
| **236** | M | 67 | 27.09 | 2 | 03.04.2020 | 08.04.2020 | 0 | 0 | 0 | 1 | 1 | 0 | 0 | 0 | 0 | 0 | 0 | 0 | P1245A |
| **237** | M | 84 | 24.32 | 3 | 03.04.2020 | 20.04.2020 | 0 | 0 | 0 | 0 | 1 | 0 | 1 | 1 | 1 | 1 | 0 | 0 | P1245A |
| **238** | M | 74 | 23.10 | 4 | 03.04.2020 | 03.04.2020 | 0 | 0 | 0 | 1 | 1 | 0 | 0 | 0 | 1 | 0 | 1 | 0 | P1245C |
| **239** | F | 54 | 24.80 | 3 | 03.04.2020 | 14.04.2020 | 0 | 0 | 0 | 1 | 1 | 0 | 1 | 0 | 0 | 0 | 0 | 0 | P1245A |
| **240** | F | 84 | 21.58 | 3 | 03.04.2020 | 20.04.2020 | 0 | 0 | 0 | 0 | 1 | 0 | 1 | 1 | 0 | 1 | 0 | 1 | P1245A |
| **241** | F | 73 | 33.81 | 3 | 04.04.2020 | 05.05.2020 | 0 | 0 | 0 | 0 | 1 | 0 | 0 | 0 | 1 | 0 | 0 | 0 | P1245C |
| **242** | M | 85 | 27.68 | 4 | 04.04.2020 | 18.04.2020 | 0 | 0 | 0 | 1 | 0 | 0 | 0 | 0 | 1 | 1 | 0 | 0 | P1245A |
| **243** | M | 94 | 22.32 | 8 | 05.04.2020 | 11.04.2020 | 1 | 0 | 0 | 0 | 1 | 0 | 0 | 0 | 1 | 0 | 0 | 1 | P1245A |
| **244** | M | 78 | 27.58 | 4 | 06.04.2020 | 17.04.2020 | 0 | 0 | 0 | 0 | 1 | 0 | 0 | 0 | 0 | 1 | 0 | 0 | P1245A>C |
| **245** | F | 61 | 25.64 | 4 | 06.04.2020 | 20.04.2020 | 0 | 0 | 0 | 1 | 1 | 0 | 0 | 0 | 0 | 0 | 0 | 0 | P1245A |
| **246** | M | 45 | 26.47 | 4 | 07.04.2020 | 15.04.2020 | 0 | 0 | 0 | 1 | 1 | 1 | 1 | 0 | 0 | 0 | 0 | 0 | P1245A>C |
| **247** | F | 74 | 26.18 | 4 | 07.04.2020 | 15.04.2020 | 0 | 0 | 0 | 0 | 1 | 0 | 0 | 1 | 0 | 1 | 0 | 0 | P1245C |
| **248** | M | 86 | 28.01 | 4 | 07.04.2020 | 27.04.2020 | 0 | 1 | 1 | 1 | 1 | 0 | 0 | 1 | 1 | 0 | 1 | 1 | P1245A |
| **249** | M | 78 | 25.06 | 4 | 08.04.2020 | 24.04.2020 | 0 | 0 | 0 | 0 | 1 | 1 | 0 | 1 | 1 | 0 | 0 | 0 | P1245A |
| **250** | F | 77 | 32.81 | 4 | 09.04.2020 | 29.04.2020 | 0 | 0 | 0 | 1 | 1 | 1 | 1 | 0 | 0 | 1 | 1 | 0 | P1245A |
| **251** | M | 83 | 34.60 | 8 | 09.04.2020 | 11.04.2020 | 1 | 0 | 0 | 1 | 1 | 0 | 1 | 0 | 1 | 1 | 0 | 0 | P1245A>C |
| **252** | M | 68 | 26.00 | 8 | 11.04.2020 | 17.04.2020 | 1 | 0 | 0 | 1 | 1 | 0 | 0 | 1 | 0 | 1 | 0 | 0 | P1245A>C |
| **253** | F | 84 | 37.02 | 3 | 11.04.2020 | 23.04.2020 | 0 | 0 | 0 | 0 | 1 | 0 | 1 | 0 | 1 | 0 | 1 | 0 | P1245A |
| **254** | F | 72 | 29.75 | 8 | 12.04.2020 | 27.04.2020 | 1 | 1 | 13 | 1 | 1 | 0 | 1 | 0 | 0 | 0 | 0 | 0 | P1245A>C |
| **255** | F | 63 | 19.06 | 3 | 13.04.2020 | 23.04.2020 | 0 | 0 | 0 | 0 | 1 | 0 | 0 | 0 | 0 | 0 | 0 | 0 | P1245A>C |
| **256** | M | 51 | 28.43 | 3 | 13.04.2020 | 15.04.2020 | 0 | 0 | 0 | 1 | 1 | 0 | 0 | 0 | 0 | 0 | 0 | 0 | P1245A>C |
| **257** | M | 86 | 30.80 | 4 | 14.04.2020 | 16.04.2020 | 0 | 0 | 0 | 1 | 1 | 0 | 1 | 1 | 1 | 1 | 0 | 0 | P1245A>C |
| **258** | M | 83 | 31.91 | 4 | 16.04.2020 | 21.04.2020 | 0 | 0 | 0 | 0 | 1 | 0 | 0 | 0 | 1 | 1 | 0 | 0 | P1245A |
| **259** | M | 52 | 32.18 | 4 | 16.04.2020 | 19.04.2020 | 0 | 0 | 0 | 1 | 1 | 0 | 1 | 0 | 0 | 1 | 0 | 0 | P1245A |
| **260** | F | 92 | 17.26 | 3 | 17.04.2020 | 20.04.2020 | 0 | 0 | 0 | 0 | 0 | 0 | 0 | 0 | 1 | 0 | 1 | 0 | P1245A |
| **261** | M | 71 | 28.95 | 4 | 17.04.2020 | 25.04.2020 | 0 | 0 | 0 | 1 | 0 | 1 | 0 | 0 | 1 | 1 | 1 | 0 | P1245A |
| **262** | M | 82 | 26.30 | 4 | 18.04.2020 | 19.04.2020 | 0 | 0 | 0 | 1 | 1 | 0 | 1 | 0 | 1 | 0 | 0 | 0 | P1245A |
| **263** | F | 75 | 23.70 | 3 | 19.04.2020 | 19.04.2020 | 0 | 0 | 0 | 1 | 0 | 1 | 1 | 0 | 0 | 0 | 0 | 0 | P1245A>C |
| **264** | M | 64 | 30.39 | 7 | 21.04.2020 | 09.05.2020 | 0 | 1 | 28 | 1 | 1 | 1 | 1 | 0 | 0 | 0 | 0 | 0 | P1245A |
| **265** | M | 78 | 30.11 | 8 | 10.03.2020 | 02.04.2020 | 1 | 1 | 20 | 0 | 1 | 1 | 0 | 0 | 0 | 0 | 0 | 1 | P1245A |
| **266** | M | 78 | NA | 4 | 20.03.2020 | 28.03.2020 | 0 | 0 | 0 | 0 | 0 | 0 | 1 | 1 | 0 | 1 | 0 | 0 | P1245A>C |
| **267** | F | 78 | 25.30 | 4 | 25.03.2020 | 05.04.2020 | 0 | 0 | 0 | 1 | 0 | 1 | 1 | 0 | 0 | 1 | 0 | 0 | P1245A |
| **268** | M | 61 | 25.90 | 6 | 24.03.2020 | 15.04.2020 | 0 | 1 | 7 | 1 | 1 | 0 | 1 | 0 | 1 | 0 | NA | 0 | P1245A>C |
| **269** | F | 76 | 30.10 | 8 | 20.03.2020 | 23.03.2020 | 1 | 1 |  | 1 | 1 | 0 | 1 | 0 | 1 | 1 | 0 | 0 | P1245A |
| **270** | F | 88 | 41.10 | 4 | 17.03.2020 | 03.04.2020 | 0 | 0 | 0 | 1 | 1 | 0 | 1 | 0 | 1 | 1 | 0 | 0 | P1245A>C |
| **271** | F | 59 | 18.60 | 3 | 26.03.2020 | 04.04.2002 | 0 | 0 | 0 | 1 | 1 | 1 | 1 | 0 | 0 | 0 | 0 | 0 | P1245A>C |
| **272** | M | 79 | 29.50 | 8 | 11.04.2020 | 21.04.2020 | 1 | 0 | 0 | 1 | 1 | 0 | 1 | 0 | 0 | 1 | 0 | 0 | P1245A>C |
| **273** | M | 72 | 26.1 | 8 | 29.03.2020 | 09.04.2020 | 1 | 1 | 31 | 0 | 0 | 0 | 1 | 0 | 0 | 0 | 1 | 0 | P1245A |
| **274** | F | 54 | 17.60 | 3 | 28.03.2020 | 02.04.2020 | 0 | 0 | 0 | 1 | 1 | 0 | 1 | 0 | 0 | 0 | 0 | 0 | P1245A>C |
| **275** | M | 73 | 29.70 | 3 | 18.03.2020 | 25.03.2020 | 0 | 0 | 0 | 0 | 0 | 0 | 1 | 1 | 0 | 1 | 0 | 1 | P1245A>C |
| **276** | F | 74 | NA | 3 | 16.03.2020 | 24.03.2020 | 0 | 0 | 0 | 1 | 1 | 0 | 1 | 0 | 0 | 1 | 1 | 0 | P1245A |
| **277** | F | 73 | NA | 3 | 25.03.2020 | 30.03.2020 | 0 | 0 | 0 | 1 | 0 | 1 | 0 | 0 | 0 | 1 | 0 | 0 | P1245A |
| **278** | F | 86 | 35.30 | 8 | 15.03.2020 | 24.03.2020 | 1 | 0 | 0 | 0 | 1 | 0 | 1 | 1 | 0 | 1 | 0 | 0 | P1245A>C |
| **279** | M | 59 | 21.90 | 3 | 07.05.2020 | 13.05.2020 | 0 | 0 | 0 | 0 | 1 | 0 | 1 | 0 | 0 | 0 | 0 | 0 | P1245A |
| **280** | M | 85 | 23.60 | 4 | 29.03.2020 | 10.04.2020 | 0 | 0 | 0 | 1 | 1 | 0 | 1 | 0 | 1 | 0 | NA | 0 | P1245A>C |
| **281** | F | 84 | 20.00 | 5 | 01.04.2020 | 10.04.2020 | 0 | 0 | 0 | 1 | 0 | 0 | 1 | 0 | 0 | 1 | NA | 1 | P1245A |
| **282** | F | 70 | NA | 4 | 25.03.2020 | 04.04.2020 | 0 | 0 | 0 | 1 | 1 | 0 | 1 | 0 | 0 | 0 | 0 | 0 | P1245A |
| **283** | F | 79 | NA | 3 | 07.04.2020 | 16.04.2020 | 0 | 0 | 0 | 1 | 1 | 1 | 1 | 0 | 0 | 0 | 0 | 0 | P1245A>C |
| **284** | M | 75 | 18.70 | 8 | 17.05.2020 | 23.05.2020 | 1 | 1 | 3 | 0 | 0 | 0 | 1 | 1 | 0 | 1 | 1 | 0 | P1245A>C |
| **285** | F | 59 | 37.10 | 4 | 18.03.2020 | 26.03.2020 | 0 | 0 | 0 | 1 | 1 | 0 | 1 | 1 | 0 | 1 | 0 | 0 | P1245A |
| **286** | F | 74 | NA | 4 | 21.03.2020 | 09.04.2020 | 0 | 0 | 0 | 0 | 0 | 0 | 1 | 0 | 0 | 0 | NA | 0 | P1245A>C |
| **287** | M | 86 | 19.60 | 8 | 07.05.2020 | 22.05.2020 | 1 | 0 | 0 | 0 | 1 | 0 | 1 | 0 | 1 | 1 | 1 | 0 | P1245A>C |
| **288** | M | 79 | NA | 4 | 19.03.2020 | 24.03.2020 | 0 | 0 | 0 | 0 | 0 | 0 | 1 | 0 | 0 | 1 | 0 | 0 | P1245A>C |
| **289** | M | 72 | 31.80 | 7 | 16.03.2020 | 28.04.2020 | 0 | 1 | 35 | 1 | 1 | 0 | 1 | 1 | 0 | 1 | 0 | 0 | P1245C |
| **290** | M | 84 | 22.80 | 4 | 16.05.2020 | 01.06.2020 | 0 | 0 | 0 | 0 | 1 | 0 | 1 | 0 | 0 | 1 | 1 | 1 | P1245A |
| **291** | F | 80 | 27.70 | 3 | 22.05.2020 | 01.06.2020 | 0 | 0 | 0 | 0 | 0 | 1 | 1 | 0 | 0 | 1 | 0 | 0 | P1245A |
| **292** | M | 70 | 22.60 | 4 | 26.03.2020 | 07.04.2020 | 0 | 0 | 0 | 1 | 1 | 0 | 1 | 0 | 0 | 0 | 0 | 1 | P1245C |
| **293** | M | 85 | 23.60 | 4 | 03.04.2020 | 11.04.2020 | 1 | 0 | 0 | 1 | 1 | 0 | 1 | 0 | 1 | 1 | NA | 0 | P1245A |
| **294** | M | 56 | 32.60 | 7 | 28.03.2020 | 05.05.2020 | 0 | 1 | 23 | 1 | 0 | 1 | 1 | 0 | 0 | 0 | 0 | 0 | P1245A |
| **295** | F | 50 | NA | 3 | 30.03.2020 | 02.04.2020 | 0 | 0 | 0 | 1 | 1 | 0 | 1 | 0 | 0 | 0 | 0 | 0 | P1245A |
| **296** | M | 53 | 22.00 | 3 | 13.04.2020 | 17.04.2020 | 0 | 0 | 0 | 0 | 0 | 0 | 1 | 0 | 0 | 1 | NA | 0 | P1245A |
| **297** | F | 59 | NA | 4 | 28.03.2020 | 01.04.2020 | 0 | 0 | 0 | 1 | 1 | 0 | 1 | 0 | 0 | 0 | 0 | 0 | P1245A |
| **298** | M | 75 | 28.40 | 8 | 25.03.2020 | 12.06.2020 | 1 | 1 | 66 | 1 | 1 | 0 | 1 | 1 | 0 | 1 | 0 | 0 | P1245A>C |
| **299** | F | 73 | NA | 4 | 25.03.2020 | 04.04.2020 | 0 | 0 | 0 | 1 | 1 | 0 | 1 | 0 | 0 | 0 | 0 | 0 | P1245A>C |
| **300** | M | 67 | 24.40 | 3 | 28.03.2020 | 05.04.2020 | 0 | 0 | 0 | 0 | 1 | 0 | 1 | 0 | 1 | 1 | 1 | 0 | P1245A |
| **301** | F | 76 | 25.60 | 3 | 23.03.2020 | 27.03.2020 | 0 | 0 | 0 | 1 | 1 | 0 | 1 | 0 | 0 | 1 | 0 | 0 | P1245A>C |
| **302** | M | 63 | 24.90 | 5 | 19.03.2020 | 08.04.2020 | 0 | 0 | 0 | 0 | 0 | 0 | 1 | 0 | 0 | 0 | NA | 0 | P1245A>C |
| **303** | F | 82 | NA | 8 | 19.03.2020 | 21.03.2020 | 1 | 0 | 0 | 1 | 0 | 0 | 1 | 1 | 0 | 0 | 0 | 0 | P1245A |
| **304** | F | 75 | 32.00 | 5 | 22.03.2020 | 06.04.2020 | 0 | 0 | 0 | 1 | 1 | 0 | 1 | 1 | 0 | 1 | NA | 0 | P1245A>C |
| **305** | M | 69 | 29.90 | 4 | 27.03.2020 | 03.04.2020 | 0 | 0 | 0 | 1 | 1 | 0 | 1 | 0 | 0 | 0 | 0 | 0 | P1245A |
| **306** | M | 67 | 26.80 | 4 | 18.03.2020 | 27.03.2020 | 0 | 0 | 0 | 1 | 1 | 0 | 1 | 0 | 0 | 1 | 0 | 0 | P1245A>C |
| **307** | M | 67 | 35.90 | 8 | 26.03.2020 | 05.04.2020 | 1 | 1 | 8 | 1 | 1 | 0 | 1 | 0 | 1 | 1 | 1 | 0 | P1245A>C |
| **308** | M | 80 | 26.90 | 8 | 20.04.2020 | 27.04.2020 | 1 | 1 | 4 | 1 | 0 | 0 | 1 | 1 | 1 | 1 | 1 | 0 | P1245C |
| **309** | M | 61 | NA | 3 | 25.03.2020 | 01.04.2020 | 0 | 0 | 0 | 0 | 1 | 0 | 1 | 1 | 1 | 0 | 0 | 1 | P1245A |
| **310** | M | 78 | 26.30 | 6 | 17.04.2020 | 07.05.2020 | 0 | 1 | 5 | NA | NA | NA | 1 | 1 | 1 | 1 | 0 | 0 | P1245C |
| **311** | F | 75 | 32.30 | 5 | 24.03.2020 | 20.04.2020 | 0 | 0 | 0 | 0 | 0 | 0 | 1 | 0 | 0 | 1 | NA | 0 | P1245A>C |
| **312** | M | 75 | 27.10 | 4 | 30.03.2020 | 09.04.2020 | 0 | 0 | 0 | 0 | 0 | 0 | 1 | 0 | 0 | 0 | NA | 0 | P1245A>C |
| **313** | F | 42 | 18.80 | 3 | 05.04.2020 | 07.04.2020 | 0 | 0 | 0 | 0 | 0 | 0 | 0 | 0 | 0 | 0 | NA | 0 | P1245C |
| **314** | M | 79 | 23.90 | 4 | 18.03.2020 | 23.03.2020 | 0 | 0 | 0 | 1 | 1 | 1 | 1 | 0 | 0 | 0 | 0 | 0 | P1245A>C |
| **315** | F | 96 | 22.40 | 3 | 31.03.2020 | 07.04.2020 | 0 | 0 | 0 | 1 | 1 | 0 | 1 | 0 | 0 | 1 | 0 | 0 | P1245A |
| **316** | M | 84 | 26.60 | 3 | 20.03.2020 | 26.03.2020 | 0 | 0 | 0 | 0 | 1 | 0 | 1 | 1 | 1 | 1 | 1 | 0 | P1245A |
| **317** | F | 72 | 22.10 | 4 | 23.03.2020 | 10.04.2020 | 0 | 0 | 0 | 0 | 0 | 0 | 1 | 0 | 0 | 0 | NA | 0 | P1245A |
| **318** | M | 89 | 20.10 | 8 | 02.05.2020 | 06.05.2020 | 1 | 0 | 0 | 0 | 0 | 0 | 1 | 0 | 1 | 0 | 0 | 0 | P1245A |
| **319** | F | 81 | NA | 3 | 23.03.2020 | 26.03.2020 | 0 | 0 | 0 | 1 | 0 | 0 | NA | 0 | 0 | 1 | 0 | 0 | P1245A |
| **320** | F | 59 | 27.40 | 4 | 25.03.2020 | 07.04.2020 | 0 | 0 | 0 | 1 | 1 | 0 | 1 | 0 | 0 | 0 | 1 | 0 | P1245A |
| **321** | M | 76 | NA | 5 | 26.03.2020 | 06.04.2020 | 0 | 0 | 0 | 1 | 1 | 0 | 1 | 1 | 1 | 1 | 1 | 0 | P1245A>C |
| **322** | M | 67 | 28.00 | 4 | 30.03.2020 | 08.04.2020 | 0 | 0 | 0 | 0 | 0 | 0 | 1 | 0 | 0 | 1 | NA | 0 | P1245A>C |
| **323** | F | 63 | NA | 4 | 18.03.2020 | 27.03.2020 | 0 | 0 | 0 | 1 | 0 | 0 | 1 | 1 | 0 | 1 | 0 | 0 | P1245A |
| **324** | M | 75 | NA | 3 | 16.04.2020 | 30.04.2020 | 0 | 0 | 0 | 0 | 0 | 0 | 1 | 0 | 0 | 0 | 0 | 0 | P1245A>C |
| **325** | M | 56 | 30.70 | 4 | 21.03.2020 | 27.03.2020 | 0 | 0 | 0 | 1 | 1 | 0 | 1 | 0 | 0 | 0 | 0 | 0 | P1245C |
| **326** | M | 71 | 27.80 | 4 | 27.03.2020 | 10.04.2020 | 0 | 0 | 0 | 1 | 1 | 0 | 1 | 0 | 1 | 1 | NA | 0 | P1245A>C |
| **327** | M | 58 | 35.90 | 3 | 21.03.2020 | 27.03.2020 | 0 | 0 | 0 | 1 | 1 | 0 | 1 | 0 | 0 | 1 | 0 | 0 | P1245A>C |
| **328** | F | 87 | 30.80 | 4 | 19.04.2020 | 04.05.2020 | 0 | 0 | 0 | 1 | 0 | 0 | 1 | 0 | 0 | 0 | 0 | 0 | P1245A>C |
| **329** | F | 53 | NA | 4 | 21.03.2020 | 02.04.2020 | 0 | 0 | 0 | 1 | 1 | 1 | 1 | 0 | 0 | 1 | 0 | 0 | P1245A>C |
| **330** | F | 75 | NA | 4 | 16.03.2020 | 28.03.2020 | 0 | 0 | 0 | 1 | 1 | 0 | 1 | 0 | 0 | 0 | 0 | 0 | P1245A |
| **331** | F | 59 | NA | 3 | 23.03.2020 | 30.03.2020 | 0 | 0 | 0 | 1 | 1 | 0 | 1 | 1 | 0 | 1 | 0 | 0 | P1245C |
| **332** | M | 44 | 30.00 | 5 | 24.03.2020 | 04.04.2020 | 0 | 0 | 0 | 1 | 1 | 0 | 1 | 0 | 0 | 0 | 1 | 0 | P1245A>C |
| **333** | M | 82 | 25.20 | 4 | 17.03.2020 | 26.03.2020 | 0 | 0 | 0 | 1 | 1 | 0 | 1 | 0 | 0 | 0 | 0 | 0 | P1245A |
| **334** | M | 81 | 22.50 | 8 | 15.04.2020 | 08.05.2020 | 1 | 0 | 0 | 0 | 0 | 0 | 1 | 0 | 1 | 0 | NA | 0 | P1245A>C |
| **335** | M | 69 | 28.20 | 7 | 22.03.2020 | 08.04.2020 | 0 | 1 | 9 | 0 | 1 | 0 | 1 | 0 | 0 | 1 | 0 | 0 | P1245A>C |
| **336** | F | 77 | NA | 4 | 31.03.2020 | 10.04.2020 | 0 | 0 | 0 | 0 | 0 | 0 | 1 | 0 | 0 | 0 | NA | 0 | P1245A>C |
| **337** | M | 68 | 24.30 | 4 | 23.03.2020 | 03.04.2020 | 0 | 0 | 0 | 1 | 1 | 0 | 1 | 0 | 0 | 0 | 0 | 0 | P1245A>C |
| **338** | M | 82 | 21.00 | 4 | 25.03.2020 | 04.04.2020 | 0 | 0 | 0 | 0 | 0 | 0 | 1 | 0 | 1 | 1 | 1 | 0 | P1245A>C |
| **339** | M | 82 | NA | 4 | 17.03.2020 | 26.03.2020 | 0 | 0 | 0 | 1 | 1 | 0 | 1 | 1 | 1 | 1 | 1 | 1 | P1245A>C |
| **340** | F | 73 | 34.90 | 4 | 09.04.2020 | 17.04.2020 | 0 | 0 | 0 | 0 | 0 | 0 | 1 | 0 | 0 | 0 | NA | 0 | P1245A>C |
| **341** | F | 74 | 27.80 | 3 | 11.04.2020 | 17.04.2020 | 0 | 0 | 0 | 1 | 0 | 0 | 1 | 1 | 0 | 1 | NA | 0 | P1245A |
| **342** | M | 55 | 27.20 | 4 | 25.03.2020 | 03.04.2020 | 0 | 0 | 0 | 1 | 1 | 0 | 1 | 0 | 0 | 0 | 0 | 1 | P1245A>C |
| **343** | F | 41 | NA | 3 | 07.04.2020 | 09.04.2020 | 0 | 0 | 0 | 0 | 0 | 0 | 1 | 0 | 0 | 0 | NA | 0 | P1245A |
| **344** | M | 65 | NA | 4 | 23.03.2020 | 04.04.2020 | 0 | 0 | 0 | 0 | 0 | 0 | 1 | 0 | 1 | 1 | 1 | 0 | P1245C |
| **345** | M | 68 | 28.50 | 8 | 17.03.2020 | 21.03.2020 | 1 | 1 | 5 | 1 | 1 | 0 | 1 | 1 | 0 | 1 | 1 | 1 | P1245A |
| **346** | M | 68 | NA | 4 | 20.03.2020 | 01.04.2020 | 0 | 0 | 0 | 1 | 1 | 0 | 1 | 1 | 0 | 1 | 1 | 0 | P1245C |
| **347** | M | 83 | 24.90 | 8 | 18.03.2020 | 24.03.2020 | 1 | 0 | 0 | 1 | 1 | 1 | 1 | 1 | 1 | 1 | 0 | 0 | P1245A>C |
| **348** | F | 61 | 28.10 | 4 | 01.04.2020 | 02.04.2020 | 0 | 0 | 0 | 1 | 1 | 0 | 1 | 0 | 0 | 1 | 0 | 0 | P1245A |
| **349** | F | 85 | 29.60 | 8 | 09.04.2020 | 29.04.2020 | 1 | 0 | 0 | 0 | 1 | 0 | 1 | 0 | 1 | 0 | NA | 0 | P1245A>C |
| **350** | M | 73 | 23.50 | 7 | 24.03.2020 | 27.05.2020 | 0 | 0 | 0 | 0 | 0 | 0 | 1 | 0 | 0 | 0 | NA | 0 | P1245A>C |
| **351** | F | 92 | 21.90 | 8 | 27.03.2020 | 30.03.2020 | 1 | 0 | 0 | 1 | 1 | 0 | 1 | 0 | 1 | 1 | 1 | 0 | P1245C |
| **352** | M | 54 | NA | 4 | 23.03.2020 | 02.04.2020 | 0 | 0 | 0 | 1 | 1 | 0 | 1 | 1 | 1 | 1 | 0 | 0 | P1245A |
| **353** | M | 68 | 33.10 | 8 | 21.03.2020 | 11.04.2020 | 1 | 1 | 22 | 1 | 1 | 0 | 1 | 1 | 0 | 1 | 0 | 0 | P1245A>C |
| **354** | M | 50 | 33.20 | 7 | 18.03.2020 | 04.04.2020 | 0 | 1 | 10 | 1 | 1 | 0 | 1 | 1 | 0 | 1 | 1 | 0 | P1245A>C |
| **355** | M | 82 | NA | 8 | 19.03.2020 | 25.03.2020 | 1 | 0 | 0 | 1 | 1 | 0 | 1 | 0 | 0 | 1 | 0 | 0 | P1245A>C |
| **356** | M | 78 | 19.60 | 8 | 26.03.2020 | 06.04.2020 | 1 | 1 | 2 | 1 | 0 | 0 | 1 | 0 | 0 | 1 | 0 | 0 | P1245A>C |
| **357** | M | 48 | 19.40 | 3 | 29.03.2020 | 10.04.2020 | 0 | 0 | 0 | 0 | 0 | 0 | 1 | 0 | 0 | 0 | NA | 1 | P1245A>C |
| **358** | F | 58 | 32.90 | 4 | 25.03.2020 | 02.04.2020 | 0 | 0 | 0 | 1 | 1 | 0 | 1 | 0 | 0 | 0 | 1 | 0 | P1245A>C |
| **359** | M | 83 | 23.20 | 3 | 22.03.2020 | 26.03.2020 | 0 | 0 | 0 | 1 | 1 | 0 | 1 | 1 | 1 | 1 | 0 | 0 | P1245A |
| **360** | M | 40 | 24.30 | 3 | 17.03.2020 | 19.03.2020 | 0 | 0 | 0 | 0 | 1 | 0 | 1 | 0 | 1 | 0 | 1 | 0 | P1245A |
| **361** | M | 66 | 27.30 | 4 | 29.03.2020 | 08.04.2020 | 0 | NA | NA | 1 | 0 | 0 | 1 | 0 | 1 | 0 | NA | 1 | P1245A>C |
| **362** | F | 53 | 25.40 | 4 | 24.03.2020 | 30.03.2020 | 0 | 0 | 0 | 1 | 1 | 0 | 1 | 0 | 0 | 0 | 0 | 0 | P1245A |
| **363** | M | 56 | 29.00 | 4 | 24.03.2020 | 06.04.2020 | 0 | 0 | 0 | 1 | 1 | 0 | 1 | 0 | 0 | 1 | 1 | 0 | P1245C |
| **364** | M | 72 | 38.30 | 8 | 19.03.2020 | 22.03.2020 | 1 | 0 | 0 | 1 | 0 | 0 | 1 | 1 | 1 | 1 | 1 | 0 | P1245A>C |
| **365** | F | 39 | 20.00 | 3 | 07.05.2020 | 14.05.2020 | 0 | 0 | 0 | 0 | 0 | 0 | 1 | 0 | 0 | 0 | NA | 0 | P1245A>C |
| **366** | M | 74 | 35.50 | 7 | 10.04.2020 | 14.04.2020 | 0 | 1 | 2 | 0 | 1 | 0 | 1 | 1 | 1 | 1 | 0 | 0 | P1245A>C |
| **367** | M | 73 | 29.40 | 8 | 28.03.2020 | 24.04.2020 | 1 | 1 | 16 | 0 | 1 | 1 | 1 | 0 | 1 | 1 | 1 | 1 | P1245A |
| **368** | M | 79 | NA | 8 | 17.03.2020 | 19.03.2020 | 1 | 1 | 3 | NA | NA | NA | 1 | 0 | 1 | 0 | 0 | 1 | P1245A |
| **369** | F | 74 | 29.70 | 7 | 19.04.2020 | 13.05.2020 | 0 | 1 | 14 | 1 | 1 | 0 | 1 | 0 | 0 | 1 | 0 | 1 | P1245A>C |
| **370** | F | 79 | 19.10 | 4 | 18.03.2020 | 30.03.2020 | 0 | 0 | 0 | 1 | 1 | 0 | 1 | 0 | 1 | 0 | 0 | 1 | P1245A>C |
| **371** | M | 50 | 36.50 | 7 | 17.03.2020 | 04.04.2020 | 0 | 1 | 13 | 1 | 1 | 0 | 1 | 1 | 0 | 1 | 0 | 0 | P1245C |
| **372** | F | 71 | 22.30 | 3 | 22.03.2020 | 09.04.2020 | 0 | 0 | 0 | 0 | 0 | 0 | 1 | 0 | 1 | 0 | NA | 0 | P1245C |
| **373** | F | 87 | 29.90 | 3 | 20.04.2020 | 28.04.2020 | 0 | 0 | 0 | 1 | 1 | 0 | 1 | 0 | 1 | 1 | 1 | 0 | P1245A |
| **374** | M | 47 | NA | 3 | 23.03.2020 | 04.04.2020 | 0 | 0 | 0 | 1 | 1 | 0 | 1 | 0 | 0 | 0 | 0 | 1 | P1245A>C |
| **375** | M | 65 | 25.10 | 4 | 11.03.2020 | 01.04.2020 | 0 | 0 | 0 | 1 | 1 | 0 | 1 | 0 | 0 | 1 | 0 | 0 | P1245A>C |
| **376** | F | 65 | NA | 3 | 25.03.2020 | 30.03.2020 | 0 | 0 | 0 | 1 | 1 | 0 | 1 | 0 | 0 | 0 | 0 | 1 | P1245A |
| **377** | M | 58 | 26.60 | 4 | 24.03.2020 | 31.03.2020 | 0 | 0 | 0 | 1 | 1 | 0 | 1 | 0 | 0 | 1 | 1 | 0 | P1245A |
| **378** | M | 67 | 32.20 | 3 | 28.03.2020 | 07.04.2020 | 0 | 0 | 0 | 0 | 1 | 1 | 1 | 0 | 1 | 1 | NA | 0 | P1245A>C |
| **379** | M | 82 | NA | 3 | 20.03.2020 | 30.03.2020 | 0 | 0 | 0 | 0 | 1 | 0 | 1 | 1 | 1 | 0 | 0 | 1 | P1245A |
| **380** | M | 73 | 29.10 | 4 | 26.03.2020 | 06.04.2020 | 0 | 0 | 0 | 1 | 1 | 0 | 1 | 0 | 1 | 1 | 0 | 1 | P1245A |
| **381** | M | 55 | 31.30 | 4 | 03.04.2020 | 09.04.2020 | 0 | 0 | 0 | 0 | 0 | 0 | 1 | 0 | 0 | 0 | NA | 0 | P1245C |
| **382** | M | 67 | 24.20 | 7 | 16.03.2020 | 15.04.2020 | 0 | 1 | NA | 1 | 1 | 0 | 1 | 0 | 1 | 0 | NA | 0 | P1245A>C |
| **383** | F | 57 | 53.50 | 4 | 26.03.2020 | 07.04.2002 | 0 | 0 | 0 | 1 | 1 | 1 | 1 | 0 | 0 | 1 | 1 | 0 | P1245A |
| **384** | F | 76 | NA | 3 | 23.03.2020 | 30.03.2020 | 0 | 0 | 0 | 1 | 1 | 0 | 1 | 0 | 1 | 1 | 0 | 0 | P1245A>C |
| **385** | F | 58 | 18.30 | 3 | 19.03.2020 | 23.03.2020 | 0 | 0 | 0 | 0 | 0 | 1 | 1 | 0 | 0 | 0 | 0 | 0 | P1245A |
| **386** | F | 67 | NA | 3 | 20.03.2020 | 26.03.2020 | 0 | 0 | 0 | 0 | 0 | 1 | 0 | 0 | 0 | 0 | 0 | 0 | P1245C |
| **387** | F | 55 | NA | 3 | 06.04.2020 | 13.04.2020 | 0 | 0 | 0 | 0 | 0 | 0 | 1 | 0 | 0 | 0 | NA | 0 | P1245A>C |
| **388** | M | 65 | 25.30 | 8 | 20.03.2020 | 30.03.2020 | 1 | 1 | 4 | 1 | 0 | 0 | 1 | 0 | 0 | 1 | 1 | 0 | P1245A>C |
| **389** | F | 70 | 23.80 | 8 | 21.03.2020 | 16.05.2020 | 1 | 1 | 21 | 1 | 1 | 0 | 1 | 0 | 0 | 0 | 0 | 1 | P1245A |
| **390** | F | 72 | NA | 4 | 23.03.2020 | 08.04.2020 | 0 | 0 | 0 | 1 | 1 | 0 | 1 | 0 | 1 | 1 | 0 | 0 | P1245A>C |
| **391** | M | 81 | 27.20 | 8 | 25.03.2020 | 05.04.2020 | 1 | 0 | 0 | 1 | 1 | 0 | 1 | 1 | 1 | 0 | 0 | 0 | P1245A |
| **392** | F | 68 | 27.70 | 4 | 21.03.2020 | 31.03.2020 | 0 | 0 | 0 | 1 | 0 | 0 | 1 | 0 | 0 | 0 | 1 | 0 | P1245A>C |
| **393** | F | 65 | 20.40 | 3 | 16.04.2020 | 20.04.2020 | 0 | 0 | 0 | 1 | 0 | 0 | 1 | 0 | 0 | 0 | 1 | 0 | P1245A |
| **394** | F | 43 | NA | 4 | 18.03.2020 | 24.03.2020 | 0 | 0 | 0 | 0 | 1 | 0 | 1 | 0 | 0 | 0 | 0 | 0 | P1245A>C |
| **395** | F | 84 | 24.80 | 8 | 27.04.2020 | 25.05.2020 | 1 | 1 | 1 | 0 | 0 | 0 | 1 | 0 | 1 | 0 | 1 | 1 | P1245A>C |
| **396** | F | 74 | NA | 4 | 20.03.2020 | 31.03.2020 | 0 | 0 | 0 | 0 | 0 | 0 | 1 | 0 | 0 | 0 | 0 | 0 | P1245C |
| **397** | F | 96 | NA | 4 | 16.04.2020 | 30.04.2020 | 0 | 0 | 0 | 1 | 1 | 0 | 1 | 0 | 1 | 1 | 0 | 1 | P1245A |
| **398** | M | 65 | 20.20 | 4 | 23.03.2020 | 04.04.2020 | 0 | 0 | 0 | 1 | 1 | 0 | 1 | 0 | 0 | 0 | 0 | 0 | P1245A |
| **399** | M | 55 | 34.30 | 4 | 30.03.2020 | 06.04.2020 | 0 | 0 | 0 | 1 | 1 | 0 | 1 | 0 | 1 | 1 | 0 | 0 | P1245A>C |
| **400** | M | 56 | 39.70 | 7 | 21.03.2020 | 06.04.2020 | 0 | 1 | 12 | 1 | 0 | 0 | 1 | 1 | 0 | 0 | 1 | 0 | P1245C |

**Supplementary Table 1.** **Anamnesis, clinical and *HSD3B1* polymorphism data of our study cohort.** Number 1 indicates yes, number 0 indicates no. The BMI and the WHO scale of COVID-19 clinical improvement were defined following standard protocols. *HSD3B1* polymorphism was subdivided in three groups: P1245A (homozygous A), P1245C (homozygous C) and P1245A>C (heterozygous). Abbreviations: BMI, body mass index; CRD, chronic respiratory disease; F, female; GI, gastrointestinal; *HSD3B1* pol, *HSD3B1* polymorphism; ICU, intensive care unit; M, male; NA, data not available; WHO, World Health Organization.
