# Supplementary material for: P1245 Polymorphic Variants of HSD3B1 Gene Confer Different Outcome in Specific Subgroups of Patients Infected With SARS-CoV-2
Source: Front Med (Lausanne). 2022 Jul 7;8:793728. doi: 10.3389/fmed.2021.793728 (PMC9302441; doi:10.3389/fmed.2021.793728)
Supplement: Supplementary file 2 [file Table_2.DOCX]

**Variables:**

Sex and WHO scale

 WHO scale
 1 2 3 4 5 6 7 8
 F 1 0 46 79 8 4 2 26
 M 1 1 42 104 10 12 13 51

*p*:0.054

Sex and Death

 Death
 0 1
 F 139 27
 M 182 52

*p*: 0.178

Sex and ICU

 ICU
 0 1
 F 150 16
 M 177 56

*p***: 0.00038**

Sex and Respiratory symptoms

 Respiratory symptoms
 0 1
 F 51 115
 M 61 171

*p*: 0.392

Sex and Fever

 Fever
 0 1
 F 53 113
 M 51 181

*p:* **0.0348**

Sex and GI symptoms

 GI symptoms
 0 1
 F 130 36
 M 199 33

*p*: 0.0711

Sex and Diabetes

 Diabetes
 0 1
 F 126 40
 M 172 62

*p*: 0.67

Sex and Cardiovascular diseases

 Cardiovascular diseases
 0 1
 F 117 49
 M 138 96

*p*: **0.0242**

Sex and Hypertension

 Hypertension
 0 1
 F 85 81
 M 107 127

*p*: 0.328

Sex and CRD

 CRD
 0 1
 F 126 26
 M 165 54

*p*: 0.107

Sex and Tumor

 Tumor
 0 1
 F 143 23
 M 196 38

*p*: 0.608

Sex and Comorbidities

 Comorbidities
 0 1
 F 50 116
 M 54 180

*p*: 0.142

Sex and Comorbidities >1

 Comorbidities >1
 0 1
 F 98 68
 M 120 114

*p*: 0.152

Sex and Pneumonia

 Pneumonia
 0 1
 F 53 112
 M 63 171

*p*: 0.311

Sex and *HSD3B1* pol

 *HSD3B1* pol
 AA AC CC
 F 75 68 23
 M 83 119 32

*p*: 0.113

Sex and BMI

 BMI
 [14,30) [30,120)
 F 91 43
 M 156 51

*p*: 0.168

Sex and WHO

 WHO
 1to4 5to8
 F 126 14
 M 148 35

*p*: **0.0349**

Sex and *HSD3B1* pol_CC_vs_AC_AA

 *HSD3B1* Pol_CC_vs_AC_AA
 AA_AC CC
 F 143 23
 M 202 32

*p*: 1

Sex and *HSD3B1* pol_AA_vs_AC_CC

 *HSD3B1* pol_AA_vs_AC_CC
 AA AC_CC
 F 91 75
 M 151 83

*p*: 0.0638

**===================================================**
WHO Scale and Death

 Death
 WHO scale 0 1
 1 2 0
 2 1 0
 3 87 1
 4 181 2
 5 18 0
 6 16 0
 7 15 0
 8 1 76

*p*: **5.31*10^-77^**

WHO Scale and ICU

 ICU
 WHO Scale 0 1
 1 2 0
 2 1 0
 3 87 1
 4 175 7
 5 16 2
 6 0 16
 7 1 14
 8 45 32

*p*: **4.3*10^-40^**

WHO Scale and Respiratory symptoms

 Respiratory symptoms
 WHO Scale 0 1
 1 1 1
 2 0 1
 3 37 51
 4 42 141
 5 4 14
 6 2 13
 7 4 11
 8 22 54

*p*: 0.0569

WHO Scale and Fever

 Fever
 WHO Scale 0 1
 1 1 1
 2 0 1
 3 30 58
 4 42 141
 5 6 12
 6 1 14
 7 3 12
 8 21 55

*p*: 0.283

WHO Scale and GI symptoms

 GI symptoms
 WHO Scale 0 1
 1 2 0
 2 1 0
 3 72 16
 4 150 33
 5 14 4
 6 12 3
 7 13 2
 8 65 11

*p*: 0.974

WHO Scale and Diabetes

 Diabetes
 WHO Scale 0 1
 1 2 0
 2 1 0
 3 71 17
 4 142 41
 5 13 5
 6 12 4
 7 8 7
 8 49 28

*p*: 0.0976

WHO Scale and Cardiovascula diseases

 Cardiovascular diseases
 WHO Scale 0 1
 1 2 0
 2 1 0
 3 58 30
 4 124 59
 5 15 3
 6 12 4
 7 12 3
 8 31 46

*p*: 0.000317

WHO Scale and Hypertension

 Hypertension
 WHO Scale 0 1
 1 1 1
 2 1 0
 3 46 42
 4 92 91
 5 9 9
 6 8 8
 7 7 8
 8 28 49

*p*: 0.499

WHO Scale and CRD

 CRD
 WHO Scale 0 1
 1 2 0
 2 1 0
 3 66 13
 4 136 36
 5 9 5
 6 14 1
 7 10 3
 8 53 22

*p*: 0.298

WHO Scale and Tumor

 Tumor
 WHO Scale 0 1
 1 1 1
 2 1 0
 3 72 16
 4 162 21
 5 17 1
 6 14 2
 7 14 1
 8 58 19

*p*: 0.0941

WHO Scale and Comorbidities

 Comorbidities
 WHO Scale 0 1
 1 0 2
 2 1 0
 3 31 57
 4 55 128
 5 3 15
 6 5 11
 7 5 10
 8 4 73

*p*: **0.00024**

WHO Scale and Comorbidities >1

 Comorbidities >1
 WHO Scale 0 1
 1 2 0
 2 1 0
 3 52 36
 4 106 77
 5 13 5
 6 11 5
 7 7 8
 8 26 51

*p*: **0.00319**

WHO Scale and Pneumonia

 Pneumonia
 WHO Scale 0 1
 1 0 2
 2 1 0
 3 30 57
 4 58 125
 5 5 13
 6 2 14
 7 2 13
 8 18 59

*p*: 0.174

WHO Scale and *HSD3B1* pol

 *HSD3B1* pol
 WHO Scale AA AC CC
 1 1 1 0
 2 1 0 0
 3 35 41 12
 4 75 78 30
 5 7 11 0
 6 4 9 3
 7 5 7 3
 8 30 40 7

*p*: 0.723

WHO Scale and BMI

 BMI
 WHO Scale [14,30) [30,120)
 1 1 1
 2 1 0
 3 53 14
 4 117 38
 5 10 6
 6 9 7
 7 6 9
 8 50 19

*p*: 0.0531

WHO Scale and *HSD3B1* pol_CC_vs_AC_AA

 *HSD3B1* pol_CC_vs_AC_AA
 WHO Scale AA_AC CC
 1 2 0
 2 1 0
 3 76 12
 4 153 30
 5 18 0
 6 13 3
 7 12 3
 8 70 7

*p*: 0.464

WHO Scale and *HSD3B1* pol_AA_vs_AC_CC

 *HSD3B1* pol_AA_vs_AC_CC
 WHO Scale AA AC_CC
 1 1 1
 2 0 1
 3 53 35
 4 108 75
 5 11 7
 6 12 4
 7 10 5
 8 47 30

*p*: 0.84

**===================================================**

Death and ICU

 ICU
 Death 0 1
 0 279 41
 1 48 31

*p*: **0.000000112**

Death and Respiratory symptoms

 Respiratory symptoms
 Death 0 1
 0 90 230
 1 22 56

*p*: 1

Death and Fever

 Fever
 Death 0 1
 0 82 238
 1 22 56

*p*: 0.748

Death and GI symptoms

 GI symptoms
 Death 0 1
 0 262 58
 1 67 11

*p*: 0.5

Death and Diabetes

 Diabetes
 Death 0 1
 0 247 74
 1 51 28

*p*: **0.0341**

Death and Cardiovascular diseases

 Cardiovascular diseases
 Death 0 1
 0 223 98
 1 32 47

*p*: **0.00000306**

Death and Hypertension

 Hypertension
 Death 0 1
 0 164 157
 1 28 51

*p*: **0.0179**

Death and CRD

 CRD
 Death 0 1
 0 237 58
 1 54 22

*p*: 0.11

Death and Tumor

 Tumor
 Death 0 1
 0 281 40
 1 58 21

*p*: **0.00315**

Death anc Comorbidities

 Comorbidities
 Death 0 1
 0 100 221
 1 4 75

*p*: **0.00000438**

Death and Comorbidities >1

 Comorbidities >1
 Death 0 1
 0 193 128
 1 25 54

*p*: **0.00000953**

Death and Pneumonia

 Pneumonia
 Death 0 1
 0 97 223
 1 19 60

*p*: 0.337

Death and *HSD3B1* pol

 *HSD3B1* pol
 Death AA AC CC
 0 127 146 48
 1 31 41 7

*p*: 0.321

Death and BMI

 BMI
 Death [14,30) [30,120)
 0 195 75
 1 52 19

*p*: 0.983

Death and WHO

 WHO
 Death 1to4 5to8
 0 271 49
 1 3 0

*p*: 1

Death and *HSD3B1* pol_CC_vs_AC_AA

 *HSD3B1* pol_CC_vs_AC_AA
 Death AA_AC CC
 0 273 48
 1 72 7

*p*: 0.22

Death and *HSD3B1* pol_AA_vs_AC_CC

 *HSD3B1* pol_AA_vs_AC_CC
 Death AA AC_CC
 0 194 127
 1 48 31

*p*: 1

**===================================================**

ICU and Respiratory symptoms

 Respiratory symptoms
 ICU 0 1
 0 100 227
 1 12 58

*p*: **0.0339**

ICU and Fever

Fever
 ICU 0 1
 0 87 240
 1 16 54

*p*: **0.618**

ICU and GI symptoms

 GI symptoms
 ICU 0 1
 0 270 57
 1 58 12

*p*: 1

ICU and Diabetes

 Diabetes
 ICU 0 1
 0 251 76
 1 46 26

*p*: **0.0343**

ICU and Cardiovascular diseases

 Cardiovascular diseases
 ICU 0 1
 0 211 116
 1 44 28

*p*: 0.681

ICU and Hypertension

 Hypertension
 ICU 0 1
 0 162 165
 1 29 43

*p*: 0.196

ICU and CRD

 CRD
 ICU 0 1
 0 239 62
 1 52 18

*p*:0.438

ICU and Tumor

 Tumor
 ICU 0 1
 0 281 46
 1 58 14

*p*: 0.33

ICU and Comorbidities

 Comorbidities
 ICU 0 1
 0 92 235
 1 12 60

*p*: 0.0631

ICU and Comorbidities>1

Comorbidities>1
 ICU 0 1
 0 185 142
 1 33 39

*p*: 0.127

ICU and Pneumonia

Pneumonia
 ICU 0 1
 0 104 222
 1 12 60

*p*: **0.015**

ICU and *HSD3B1* pol

 *HSD3B1* pol
 ICU AA AC CC
 0 127 155 45
 1 31 31 10

*p*: 0.777

ICU and BMI

 BMI
 ICU [14,30) [30,120)
 0 205 69
 1 41 25

*p*: 0.0552

ICU and WHO

 WHO
 ICU 1to4 5to8
 0 265 17
 1 8 32

*p*: **6.2*10^-33^**

ICU and *HSD3B1* pol_CC_vs_AC_AA

  *HSD3B1* pol_CC_vs_AC_AA
 ICU AA_AC CC
 0 282 45
 1 62 10

*p*: 1

ICU and *HSD3B1* pol AA_vs_AC_CC

 *HSD3B1* pol AA_vs_AC_CC
 ICU AA AC_CC
 0 200 127
 1 41 31

*p*: 0.597

**===================================================**

Respiratory Symptoms and Fever

 Fever
 Respiratory symptoms 0 1
 0 48 64
 1 56 230

*p*: **0.00000373**

Respiratory symptoms and GI symptoms

 GI symptoms
 Respiratory symptoms 0 1
 0 90 22
 1 239 47

p: 0.54

Respiratory symptoms and Diabetes

 Diabetes
 Respiratory symptoms 0 1
 0 81 31
 1 216 70

*p*: 0.595

Respiratory symptoms and Cardiovascular diseases

 Cardiovascular diseases
 Respiratory symptoms 0 1
 0 62 50
 1 193 93

*p*: **0.0315**

Respiratory symptoms and Hypertension

 Hypertension
 Respiratory symptoms 0 1
 0 60 52
 1 131 155

*p*: 0.199

Respiratory symptoms and CRD

 CRD
 Respiratory symptoms 0 1
 0 71 21
 1 218 59

*p*: 0.871

Respiratory symptoms and Tumor

 Tumor
 Respiratory symptoms 0 1
 0 89 23
 1 249 37

*p*: 0.0802

Respiratory symptoms and Pneumonia

 Pneumonia
 Respiratory symptoms 0 1
 0 37 75
 1 79 206

*p*: 0.355

Respiratory symptoms and *HSD3B1* pol

 *HSD3B1* Poli
 Respiratory symptoms AA AC CC
 0 38 57 17
 1 119 130 37

*p*: 0.366

Respiratory symptoms and BMI

 BMI
 Respiratory symptoms [14,30) [30,120)
 0 72 22
 1 174 72

*p*: 0.344

Respiratory symptoms and WHO

 WHO
 Respiratory symptoms 1to4 5to8
 0 80 10
 1 194 38

*p*: 0.309

Respiratory symptoms and *HSD3B1* pol_CC_vs_AC_AA

 *HSD3B1* pol_CC_vs_AC_AA
 Respiratory symptoms AA_AC CC
 0 95 17
 1 249 37

*p*: 0.671

Respiratory symptoms and *HSD3B1* pol_AA_vs_AC_CC

 *HSD3B1* pol_AA_vs_AC_CC
 Respiratory symptoms AA AC_CC
 0 74 38
 1 167 119

*p*: 0.195

**===================================================**

Fever and GI symptoms

 GI symptoms
 Fever 0 1
 0 83 21
 1 246 48

*p*: 0.457

Fever and Diabetes

 Diabetes
 Fever 0 1
 0 78 26
 1 219 75

*p*: 1

Fever and Cardiovascular diseases

 Cardiovascular diseases
 Fever 0 1
 0 66 38
 1 189 105

*p*: 0.975

Fever and Hypertension

 Hypertension
 Fever 0 1
 0 52 52
 1 139 155

*p*: 0.716

Fever and CRD

 CRD
 Fever 0 1
 0 61 22
 1 228 58

*p*: 0.289

Fever and Tumor

 Tumor
 Fever 0 1
 0 91 13
 1 247 47

*p*: 0.487

Fever and Comorbidities

 Comorbidities
 Fever 0 1
 0 29 75
 1 75 219

*p*: 0.731

Fever and Comorbidities >1

 Comorbidities >1
 Fever 0 1
 0 59 45
 1 159 135

*p*: 0.725

Fever and Pneumonia

 Pneumonia
 Fever 0 1
 0 33 70
 1 83 211

*p*: 0.545

Fever and *HSD3B1* pol

 *HSD3B1* pol
 Fever AA AC CC
 0 40 50 14
 1 117 137 40

*p*: 0.965

Fever and BMI

 BMI
 Fever [14,30) [30,120)
 0 66 21
 1 180 73

*p*: 0.478
Fever and WHO

 WHO
 Fever 1to4 5to8
 0 73 10
 1 201 38

*p*: 0.503

Fever and *HSD3B1* pol_CC_vs_AC_AA

 *HSD3B1* pol_CC_vs_AC_AA
 Fever AA_AC CC
 0 90 14
 1 254 40

*p*: 1

Fever and *HSD3B1* pol_AA_vs_AC_CC

 *HSD3B1* pol_AA_vs_AC_CC
 Fever AA AC_CC
 0 64 40
 1 177 117

*p*: 0.902

**===================================================**

GI symptoms and Diabetes

 Diabetes
 GI symptoms 0 1
 0 252 77
 1 45 24

*p*: 0.0684

GI symptoms and Cardiovascular diseases

 Cardiovascular diseases
 GI symptoms 0 1
 0 212 117
 1 43 26

*p*: 0.845

GI symptoms and Hypertension

 Hypertension
 GI symptoms 0 1
 0 163 166
 1 28 41

*p*: 0.221

GI symptoms and CRD

 CRD
 GI symptoms 0 1
 0 228 73
 1 61 7

*p*: 0.0183

GI symptoms and Tumor

 Tumor
 GI symptoms 0 1
 0 276 53
 1 62 7

*p*: 0.283

GI symptoms and Comobridities

 Comorbidities
 GI symptoms 0 1
 0 85 244
 1 19 50

*p*: 0.887

GI symptoms and Comorbidities >1

 Comorbidities >1
 GS symptoms 0 1
 0 183 146
 1 35 34

*p*: 0.542

GI symptoms and Pneumonia

 Pneumonia
 GI symptoms 0 1
 0 91 237
 1 25 44

*p*: 0.206

GI symptoms and *HSD3B1* pol

 *HSD3B1* pol
 GI symptoms AA AC CC
 0 125 159 45
 1 32 28 9

*p*: 0.414

GI symptoms and BMI

 BMI
 GI symptoms [14,30) [30,120)
 0 207 75
 1 39 19

*p*: 0.427

GI symptoms and WHO

 WHO
 GI symptoms 1to4 5to8
 0 225 39
 1 49 9

*p*: 1

GI symptoms and *HSD3B1* pol_CC_vs_AC_AA

 *HSD3B1* pol_CC_vs_AC_AA
 GI symptoms AA_AC CC
 0 284 45
 1 60 9

*p*: 1

GI symptoms and *HSD3B1* pol_AA_vs_AC_CC

 *HSD3B1* pol_AA_vs_AC_CC
 GI symptoms AA AC_CC
 0 204 125
 1 37 32

*p*: 0.246

**===================================================**

Diabetes and Cardiovascular diseases

 Cardiovascular diseases
 Diabetes 0 1
 0 206 92
 1 49 53

*p*: **0.000212**

Diabetes and Hypertension

 Hypertension
 Diabetes 0 1
 0 169 129
 1 23 79

*p*: **0.00000000504**

Diabetes and CRD

 CRD
 Diabetes 0 1
 0 220 51
 1 71 29

*p*: **0.0484**

Diabetes and Tumor

 Tumor
 Diabetes 0 1
 0 256 42
 1 83 19

*p*: .0347

Diabetes and Pneumonia

 Pneumonia
 Diabetes 0 1
 0 83 214
 1 33 69

p: 0.472

Diabetes and *HSD3B1* pol

 *HSD3B1* pol
 Diabetes AA AC CC
 0 118 142 38
 1 40 45 17

*p*: 0.591

Diabetes and BMI

 BMI
 Diabetes [14,30) [30,120)
 0 194 59
 1 53 35

*p*: **0.00456**

Diabetes and WHO

 WHO
 Diabetes 1to4 5to8
 0 216 33
 1 58 16

*p*: 0.115

Diabetes and *HSD3B1* pol_CC_vs_AC_AA

 *HSD3B1* pol_CC_vs_AC_AA
 Diabetes AA_AC CC
 0 260 38
 1 85 17

*p*: 0.41

Diabetes and *HSD3B1* pol_AA_vs_AC_CC

 *HSD3B1* pol_AA_vs_AC_CC
 Diabetes AA AC_CC
 0 180 118
 1 62 40

*p*: 1

**===================================================**

Cardiovascular diseases and Hypertension

 Hypertension
 Cardiovascular diseases 0 1
 0 144 111
 1 48 97

*p*: **0.0000112**

Cardiovascular diseases and CRD

 CRD
 Cardiovascular diseases 0 1
 0 198 38
 1 93 42

*p*: **0.00115**

Cardiovascular diseases and Tumors

 Tumors
 Cardiovascular diseases 0 1
 0 224 31
 1 115 30

*p*: **0.0326**

Cardiovascular diseases and Pneumonia

 Pneumonia
 Cardiovascular diseases 0 1
 0 69 185
 1 47 98

*p*: 0.319

Cardiovascular diseases and *HSD3B1* pol

 *HSD3B1* pol
 Cardiovascular diseases AA AC CC
 0 97 120 38
 1 61 67 17

*p*: 0.585

Cardiovascular diseases and BMI

 BMI
 Cardiovascular diseases [14,30) [30,120)
 0 152 57
 1 95 37

*p*: 0.978

Cardiovascula diseases and WHO

 WHO
 Cardiovascular diseas 1to4 5to8
 0 185 39
 1 89 10

*p*: 0.128

Cardiovascular diseases and *HSD3B1* pol_CC_vs_AC_AA

 *HSD3B1* pol_CC_vs_AC_AA
 Cardiovascular disease AA_AC CC
 0 217 38
 1 128 17

*p*: 0.462

Cardiovascular diseases and *HSD3B1* pol_AA_vs_AC_CC

 *HSD3B1* pol_AA_vs_AC_CC
 Cardiovascular diseases AA AC_CC
 0 158 97
 1 84 61

*p*: 0.493
 **===================================================**

Hypertension and CRD

 CRD
 Hypertension 0 1
 0 142 30
 1 149 50

*p*: 0.0953

Hypertension and Tumor

 Tumor
 Hypertension 0 1
 0 162 30
 1 177 31

*p*: 0.951

Hypertension and Pneumonia

 Pneumonia
 Hypertension 0 1
 0 56 136
 1 60 147
*p*: 1

Hypertension and *HSD3B1* pol

 *HSD3B1* pol
 Hypertension AA AC CC
 0 76 91 25
 1 82 96 30

*p*: 0.916

Hypertension and BMI

 BMI
 Hypertension [14,30) [30,120)
 0 124 35
 1 123 59

*p*: **0.043**

Hypertension and WHO

 WHO
 Hypertension 1to4 5to8
 0 140 24
 1 134 25

*p*: 0.906

Hypertension and *HSD3B1* pol_CC_vs_AC_AA

 *HSD3B1* pol_CC_vs_AC_AA
 Hypertension AA_AC CC
 0 167 25
 1 178 30

*p*: 0.794

Hypertension and *HSD3B1* pol_AA_vs_AC_CC

 *HSD3B1* pol_AA_vs_AC_CC
 Hypertension AA AC_CC
 0 116 76
 1 126 82

*p*: 1

**===================================================**

CRD and Tumor

 Tumor
 CRD 0 1
 0 250 41
 1 63 17

*p*: 0.165

CRD and Pneumonia

 Pneumonia
 CRD 0 1
 0 93 197
 1 22 58

*p*: 0.519

CRD and *HSD3B1* pol

 *HSD3B1* pol
 CRD AA AC CC
 0 115 136 40
 1 37 31 12

*p*: 0.438

CRD and BMI

 BMI
 CRD [14,30) [30,120)
 0 174 69
 1 53 20

*p*: 0.986

CRD and WHO

 WHO
 CRD 1to4 5to8
 0 205 33
 1 49 9

*p*: 0.91

CRD and *HSD3B1* pol_CC_vs_AC_AA

 *HSD3B1* pol_CC_vs_AC_AA
 CRD AA_AC CC
 0 251 40
 1 68 12

*p*: 0.917

CRD and *HSD3B1* pol_AA_vs_AC_CC

 *HSD3B1* pol_AA_vs_AC_CC
 CRD AA AC_CC
 0 176 115
 1 43 37

*p*: 0.339

**===================================================**

Tumor and Pneumonia

 Pneumonia
 Tumor 0 1
 0 99 239
 1 17 44

*p*: 0.943

Tumor and *HSD3B1* pol

 *HSD3B1* pol
 Tumor AA AC CC
 0 130 157 52
 1 28 30 3

*p*: 0.0854

Tumor and BMI

 BMI
 Tumor [14,30) [30,120)
 0 206 81
 1 41 13

*p*: 0.646
Tumor and WHO

 WHO
 Tumor 1to4 5to8
 0 236 45
 1 38 4

*p*: 0.388
Tumor and *HSD3B1* pol_CC_vs_AC_AA

 *HSD3B1* pol_CC_vs_AC_AA
 Tumor AA_AC CC
 0 287 52
 1 58 3

*p*: **0.0484**

Tumor and *HSD3B1* pol_AA_vs_AC_CC

 *HSD3B1* pol_AA_vs_AC_CC
 Tumor AA AC_CC
 0 209 130
 1 33 28

*p*: 0.333

**===================================================**

Comorbidities and Pneumonia

 Pneumonia
 Comorbidities 0 1
 0 28 76
 1 88 207

*p*: 0.663

Comorbidities and *HSD3B1* pol

 *HSD3B1* pol
 Comorbidities AA AC CC
 0 40 50 14
 1 118 137 41

*p*: 0.951

Comorbidities and BMI

 BMI
 Comorbidities [14,30) [30,120)
 0 63 19
 1 184 75

*p*: 0.379

Comorbidities and WHO

 WHO
 Comorbidities 1to4 5to8
 0 87 13
 1 187 36

*p*: 0.575

Comorbidities and *HSD3B1* pol_CC_vs_AC_AA

*HSD3B1* pol_CC_vs_AC_AA
 Comorbidities AA_AC CC
 0 90 14
 1 255 41

*p*: 1

Comorbidities and *HSD3B1* pol_AA_vs_AC_CC

 *HSD3B1* pol_AA_vs_AC_CC
 Comorbidities AA AC_CC
 0 64 40
 1 178 118

*p*: 0.892

**===================================================**

Comorbidities >1 and Pneumonia

 Pneumonia
 Comorbidities >1 0 1
 0 58 159
 1 58 124

*p*: 0.31

Comorbidities >1 and *HSD3B1* pol

 *HSD3B1* pol
 Comorbidities >1 AA AC CC
 0 83 104 31
 1 75 83 24

*p*: 0.812

Comorbidities >1 and BMI

 BMI
 Comorbidities >1 [14,30) [30,120)
 0 137 40
 1 110 54

*p*: **0.0443**

Comorbidities >1 and WHO

 WHO
 Comorbidities >1 1to4 5to8
 0 161 31
 1 113 18

*p*: 0.664

Comorbidities >1 and *HSD3B1* pol_CC_vs_AC_AA

 *HSD3B1* pol_CC_vs_AC_AA
 Comorbidities >1 AA_AC CC
 0 187 31
 1 158 24

*p*: 0.878

Comorbidities >1 and *HSD3B1* pol_AA_vs_AC_CC

 *HSD3B1* pol_AA_vs_AC_CC
 Comorbidities >1 AA AC_CC
 0 135 83
 1 107 75

*p*: 0.592

**===================================================**

Pneumonia and *HSD3B1* pol

 *HSD3B1* pol
 Pneumonia AA AC CC
 0 53 44 19
 1 104 143 36

*p*: 0.0722

Pneumonia and BMI

 BMI
 Pneumonia [14,30) [30,120)
 0 82 26
 1 165 68

*p*: 0.394

Pneumonia and WHO

 WHO
 Pneumonia 1to4 5to8
 0 89 9
 1 184 40

*p*: 0.068

Pneumonia and *HSD3B1* pol_CC_vs_AC_AA

 *HSD3B1* pol_CC_vs_AC_AA
 Pneumonia AA_AC CC
 0 97 19
 1 247 36

*p*: 0.422

Pneumonia and *HSD3B1* pol_AA_vs_AC_CC

 *HSD3B1* pol_AA_vs_AC_CC
 Pneumonia AA AC_CC
 0 63 53
 1 179 104

*p*: 0.122

**===================================================**
*HSD3B1* pol and BMI

 BMI
 *HSD3B1* pol [14,30) [30,120)
 AA 97 33
 AC 118 47
 CC 32 14

*p*: 0.752

*HSD3B1* pol and WHO

 WHO
 *HSD3B1* pol 1to4 5to8
 AA 112 16
 AC 120 27
 CC 42 6

*p*: 0.343

**===================================================**
BMI and WHO

 WHO
 BMI 1to4 5to8
 [14,30) 172 25
 [30,120) 53 22

*p*: **0.00218**

BMI and *HSD3B1* pol_CC_vs_AC_AA

 *HSD3B1* pol_CC_vs_AC_AA
 BMI AA_AC CC
 [14,30) 215 32
 [30,120) 80 14

*p*: 0.771

BMI and *HSD3B1* pol_AA_vs_AC_CC

  *HSD3B1* pol_AA_vs_AC_CC
 BMI AA AC_CC
 [14,30) 150 97
 [30,120) 61 33

*p*: 0.56

**===================================================**

WHO and *HSD3B1* pol_CC_vs_AC_AA

 *HSD3B1* pol_CC_vs_AC_AA
 WHO AA_AC CC
 1to4 232 42
 5to8 43 6

*p*: 0.733

WHO and *HSD3B1* pol_AA_vs_AC_CC

 *HSD3B1* pol_AA_vs_AC_CC
 WHO AA AC_CC
 1to4 162 112
 5to8 33 16

*p*: 0.355

**Supplementary Table 2. Chi-square test of the bivariate marginal distributions**. Number 1 indicates yes, number 0 indicates no. The BMI and the WHO scale of COVID-19 clinical improvement were defined following standard protocols. *HSD3B1* polymorphism was subdivided in three groups: AA (homozygous A), AC (heterozygous) and CC (homozygous C). Abbreviations: BMI, body mass index; CRD, chronic respiratory disease; F, female; GI symptoms, gastrointestinal symptoms; *HSD3B1* pol, *HSD3B1* polymorphism; ICU, intensive care unit; M, male; WHO, World Health Organization.
