# Supplementary material for: P1245 Polymorphic Variants of HSD3B1 Gene Confer Different Outcome in Specific Subgroups of Patients Infected With SARS-CoV-2
Source: Front Med (Lausanne). 2022 Jul 7;8:793728. doi: 10.3389/fmed.2021.793728 (PMC9302441; doi:10.3389/fmed.2021.793728)
Supplement: Supplementary file 3 [file Table_3.DOCX]

A.

| **Comorbidity** | ***HSD3B1* pol** | | | *p* value |
| --- | --- | --- | --- | --- |
|  | P1245A | P1245A>C | P1245C |  |
| **Diabetes** |  | | | 0.591 |
| 0 | 118 | 142 | 38 |  |
| 1 | 40 | 45 | 17 |  |
| **Cardiovascular diseases** |  | | | 0.585 |
| 0 | 97 | 120 | 38 |  |
| 1 | 61 | 67 | 17 |  |
| **Hypertension** |  | | | 0.916 |
| 0 | 76 | 91 | 25 |  |
| 1 | 82 | 96 | 30 |  |
| **CRD** |  | | | 0.438 |
| 0 | 115 | 136 | 40 |  |
| 1 | 37 | 31 | 12 |  |
| **Tumor** |  | | | 0.0854 |
| 0 | 130 | 157 | 52 |  |
| 1 | 28 | 30 | 3 |  |

B.

| **Comorbidity** | ***HSD3B1* pol** | | *p* value |
| --- | --- | --- | --- |
|  | P1245C | P1245A + P1245A>C |  |
| **Diabetes** |  | | 0.41 |
| 0 | 38 | 260 |  |
| 1 | 17 | 85 |  |
| **Cardiovascular diseases** |  | | 0.462 |
| 0 | 38 | 217 |  |
| 1 | 17 | 128 |  |
| **Hypertension** |  | | 0.794 |
| 0 | 25 | 167 |  |
| 1 | 30 | 178 |  |
| **CRD** |  | | 0.917 |
| 0 | 40 | 251 |  |
| 1 | 12 | 68 |  |
| **Tumor** |  | | **0.0484** |
| 0 | 52 | 287 |  |
| 1 | 3 | 58 |  |

C.

| **Comorbidity** | ***HSD3B1* pol** | | *p* value |
| --- | --- | --- | --- |
|  | P1245A | P1245C + P1245A>C |  |
| **Diabetes** |  | | 1 |
| 0 | 180 | 118 |  |
| 1 | 62 | 40 |  |
| **Cardiovascular diseases** |  | | 0.493 |
| 0 | 97 | 158 |  |
| 1 | 61 | 84 |  |
| **Hypertension** |  | | 1 |
| 0 | 116 | 76 |  |
| 1 | 126 | 82 |  |
| **CRD** |  | | 0.339 |
| 0 | 176 | 115 |  |
| 1 | 43 | 37 |  |
| **Tumor** |  | | 0.333 |
| 0 | 209 | 130 |  |
| 1 | 33 | 28 |  |

**Supplementary Table 3. Distribution of P1245A, P1245A>C and P1245C in each co-morbidity subgroup. Statistical significance was evaluated applying Chi-square test**. A. Evaluation of the three different HSD3B1 polymorphic variants; B. Evaluation of A allele contribution (P1245A+ P1245A>C) versus the homozygous C variant (P1245C): C. Evaluation of C allele contribution (P1245C + P1245A>C) versus the homozygous A variant (P1245A). Abbreviations: CRD, cronic respiratory disease; HSD3B1 pol, HSD3B1 polymorphism; P1245A (homozygous A), P1245C (homozygous C) and P1245A>C (heterozygous).
