# Supplementary figures and images for: P1245 Polymorphic Variants of HSD3B1 Gene Confer Different Outcome in Specific Subgroups of Patients Infected With SARS-CoV-2
Source: Front Med (Lausanne). 2022 Jul 7;8:793728. doi: 10.3389/fmed.2021.793728 (PMC9302441; doi:10.3389/fmed.2021.793728)

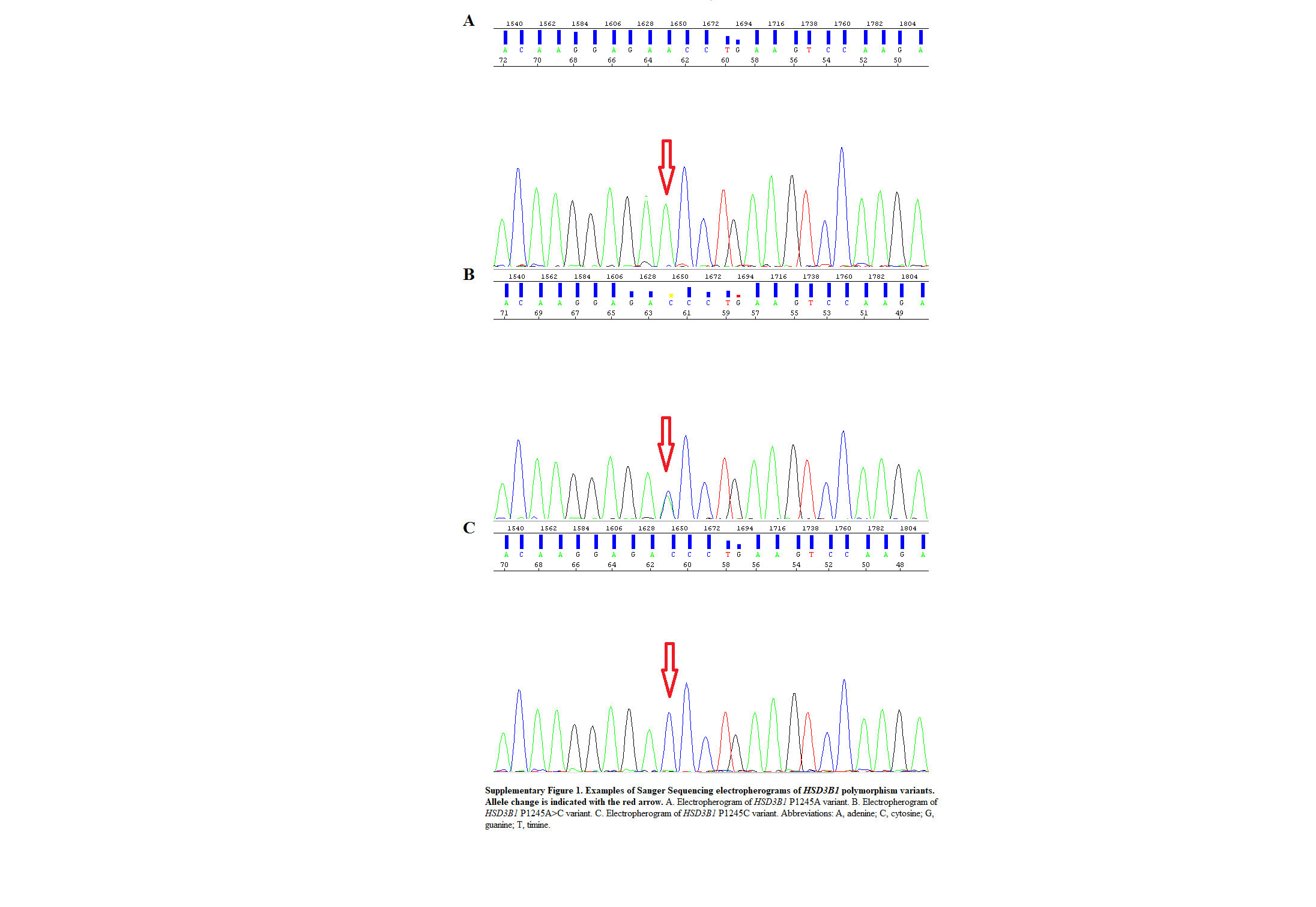

Supplement: Supplementary file 4 [file Image_1.TIF]
